# RIG-I–dependent sensing of PEDV shapes epithelial antiviral immunity in the intestinal mucosa

**DOI:** 10.1128/jvi.00483-26

**Published:** 2026-06-16

**Authors:** Hui Zeng, Yuxuan Shi, Qiu Zhong, Rongfeng Tang, Yunlei Cao, Wenqian Wang, Xinyu Miao, Xiaolin Zhang, Tao Qin, Yuchen Li, Qian Yang

**Affiliations:** 1 MOE Joint International Research Laboratory of Animal Health and Food Safety, College of Veterinary Medicine, Nanjing Agricultural University 261674, Nanjing, Jiangsu, China; 2 College of Veterinary Medicine, Yangzhou University 614704, Yangzhou, China; University of North Carolina at Chapel Hill, Chapel Hill, North Carolina, USA

**Keywords:** RIG-I, PEDV, viral PAMP, RNA secondary structure, RNA sensing, innate immune activation

## Abstract

Porcine epidemic diarrhea virus (PEDV) is a highly contagious enteric coronavirus that causes lethal diarrhea in neonatal piglets, yet the viral pathogen-associated molecular patterns (PAMPs) that initiate mucosal antiviral immunity remain poorly defined. Here, integrating intestinal single-cell transcriptomics with *in vivo* and *in vitro* infection models, we delineate how PEDV is sensed by host innate immunity. We found that RIG-I, rather than MDA5, functions as the dominant cytosolic sensor that detects PEDV and initiates interferon-mediated antiviral defense in the intestinal mucosa. RIP-seq and RNA structural analyses identified nucleotides 375-760 within the 5′ ORF1a region of the PEDV genome as a core RNA PAMP directly engaged by RIG-I. Molecular docking and mutational analyses further revealed that specific nucleotide motifs and cooperative stem-loop assemblies are required for optimal RIG-I activation, establishing higher-order RNA architecture as a key determinant of PAMP potency. Functionally, this PEDV-derived RNA PAMP robustly activated RIG-I signaling, amplified interferon and ISG responses, and restricted replication of PEDV and multiple heterologous RNA viruses, demonstrating broad-spectrum activity against several enteric and respiratory RNA viruses. Moreover, co-administration of this viral RNA with an inactivated influenza vaccine significantly enhanced antigen-specific immunity and protection. Together, these findings define the molecular basis of RIG-I–mediated recognition of PEDV, identify a structurally encoded viral PAMP with both antiviral and adjuvant properties, and provide new insights into how coronaviruses are sensed and controlled at mucosal surfaces.

**IMPORTANCE:**

Porcine epidemic diarrhea virus (PEDV) causes devastating enteric disease in newborn piglets, yet how the intestinal mucosa detects this coronavirus and mounts antiviral immunity has remained unclear. Our study identifies RIG-I as the dominant epithelial sensor responsible for detecting PEDV and initiating interferon-driven antiviral defense. We further define a structurally encoded RNA element within the 5′ ORF1a region of the PEDV genome that functions as a core viral PAMP directly activating RIG-I. This RNA element acts as a molecular alarm that triggers robust innate immune responses, restricts infection by PEDV and other RNA viruses, and enhances the protective efficacy of an inactivated influenza vaccine. These findings illuminate fundamental mechanisms of coronavirus sensing at mucosal surfaces and highlight viral RNA structures as promising natural immunostimulants for antiviral and vaccine strategies.

## INTRODUCTION

The recurrent emergence of viral infectious diseases poses a persistent threat to global health and livestock production, causing repeated epidemics and substantial economic losses (1). Evidence indicates that in acute viral infections, early pathogen recognition and prompt restriction of replication by the innate immune system are decisive determinants of disease outcome (2). Innate recognition relies on pattern recognition receptors (PRRs), which detect pathogen-associated molecular patterns (PAMPs) exposed during viral invasion and rapidly activate antiviral signaling pathways. This leads to the production of interferons and proinflammatory cytokines, thereby establishing a broad and effective antiviral state (3). In recent years, multiple PRR families have been shown to play crucial roles in viral recognition, including Toll-like receptors (TLRs) at the plasma or endosomal membranes, RIG-I-like receptors (RLRs) in the cytosol, and DNA sensors such as cGAS (4, 5). These receptors converge on signaling cascades, such as TBK1–IRF3/7 and IKK–NF-κB, to drive the transcription of type I interferons (IFN-I) and inflammatory cytokines (6), which in turn induce interferon-stimulated genes (ISGs) and activate effector immune cells to inhibit viral replication at multiple stages (7, 8). Thus, delineating the modes of action and recognition mechanisms of distinct PRRs is essential for understanding early host–virus interplay and for informing antiviral strategies and vaccine design.

RNA viruses, characterized by high mutation rates and frequent recombination, readily evade immune surveillance and cross species barriers, making them major drivers of emerging and re-emerging infectious diseases worldwide. Early recognition of RNA viruses depends primarily on two families of PRRs: TLRs and RLRs. TLR3, expressed in dendritic cells and epithelial cells, detects double-stranded RNA produced during viral replication, whereas TLR7 and TLR8 sense uridine-rich single-stranded RNA in immune cells, such as dendritic cells, macrophages, and B cells, with plasmacytoid dendritic cells serving as the dominant source of TLR7 expression (9). These TLR-mediated pathways initiate type I interferon production and local antiviral responses. By contrast, RLRs—including retinoic acid-inducible gene I (RIG-I), melanoma differentiation-associated protein 5 (MDA5), and laboratory of genetics and physiology 2 (LGP2)—are broadly expressed in almost all nucleated cells, where they recognize viral genomic RNA, replication intermediates, and terminal structures in the cytoplasm, enabling the rapid induction of type I interferons and proinflammatory cytokines at the earliest stage of infection (10, 11). Functionally, TLRs and RLRs act in a complementary manner: TLRs primarily sense extracellular or endosomal RNAs, whereas RLRs specifically monitor replication-derived RNAs in the cytoplasm (12, 13). Because RNA virus replication and transcription largely occur in the cytoplasm (14, 15), RLRs are considered the central mediators of early recognition and indispensable sentinels of host antiviral defense. Within this family, RIG-I preferentially detects 5′-triphosphate RNA and short double-stranded RNA, whereas MDA5 responds to long double-stranded RNA, together ensuring surveillance of diverse RNA signatures. Although LGP2 lacks CARD domains required for direct signaling, it fine-tunes RIG-I and MDA5 activity and helps maintain immune homeostasis (16). Collectively, this division of labor establishes RLRs as the principal drivers of early RNA virus recognition and positions them as critical molecular sentinels in the host’s frontline antiviral barrier.

Although RLRs are well recognized as key drivers of interferon responses through MAVS signaling during infections with RNA viruses such as dengue, Zika, and influenza (17–19), the structural principles governing their RNA binding and activation remain poorly understood. Viral RNAs display extensive sequence and structural heterogeneity—including long double-stranded segments, internal loops, bulges, and polyuridine tracts—that varies not only across viral species but also among RNA molecules within a single virus (20). Such diversity suggests that RLR recognition cannot be explained by a simple “lock-and-key” model but instead relies on multilevel structural cooperation and dynamic conformational regulation. The complexity is further amplified in mucosal infection, where viruses encounter a barrier composed of epithelial cells, dendritic cells, macrophages, and lymphocytes, creating a highly heterogeneous immune landscape. Expression patterns and response magnitudes of RLRs differ across these cell types, implying that mucosal recognition operates through cell type-specific programs. For example, intestinal epithelial cells, as the primary sites of viral replication, directly influence the strength of the early interferon response, whereas dendritic cells and macrophages predominantly amplify signaling and coordinate cross-talk. Elucidating how RLRs function within this mucosal context is therefore critical not only for understanding RNA virus pathogenesis at barrier surfaces but also for guiding the design of antiviral strategies that harness mucosal immunity.

Porcine epidemic diarrhea virus (PEDV), one of the most virulent enteric alphacoronaviruses, continues to threaten the global swine industry (21). PEDV preferentially infects the small intestinal epithelium of neonatal piglets, causing acute watery diarrhea and severe dehydration, with case fatality rates approaching 100% in the absence of maternal antibodies (22). Despite progress in vaccine development and herd management over the past decade, rapid viral evolution, immune evasion, and ongoing circulation within pig populations have sustained recurrent outbreaks and endemic persistence. Mounting evidence indicates that innate immune responses in the intestinal mucosa, particularly PRR-mediated interferon signaling, are central to early viral recognition, clearance, and disease progression during PEDV infection (23, 24). Yet, the dominant PRR responsible for sensing PEDV in the porcine gut, the nature of the critical PAMPs it detects, and the mechanisms by which these signals are amplified into systemic antiviral responses remain unresolved. Preliminary analyses using single-cell RNA sequencing and fluorescence *in situ* hybridization revealed a strong association between RIG-I transcriptional activity and PEDV infection. Building on these observations, this study aims to define the essential role of RIG-I in early PEDV recognition and antiviral signaling, delineate the molecular mechanisms underlying its detection of viral PAMPs, and characterize the structural features of the PEDV genome that serve as its ligands. These insights will not only advance understanding of how the host innate immune system senses PEDV and how the virus evades surveillance but also identify potential molecular targets to overcome the limitations of current countermeasures and enable the development of PRR-based precision strategies for PEDV control.

## RESULTS

### PEDV infection selectively activates the RIG-I signaling pathway in intestinal epithelial cells

**Fig 1:**
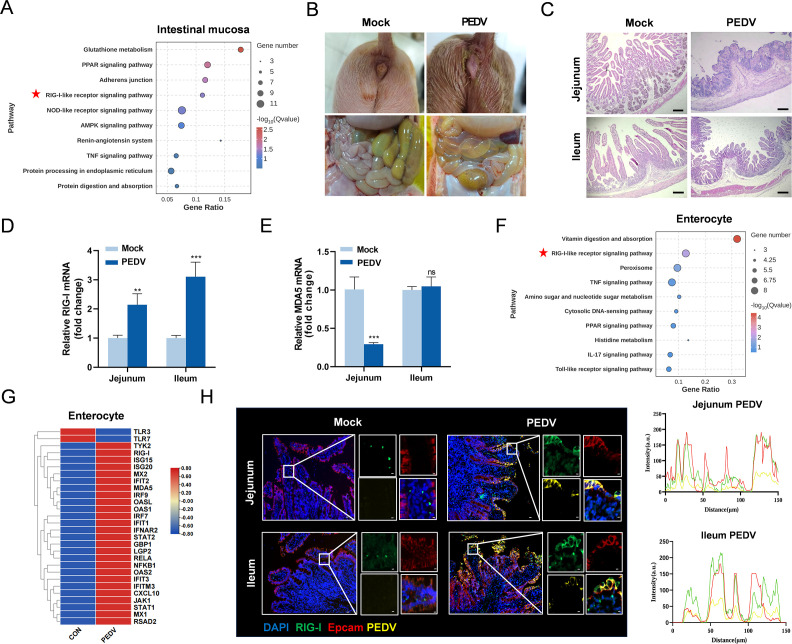
RIG-I is strongly induced in the intestinal mucosa following oral PEDV infection. (**A**) KEGG enrichment of differentially expressed genes in intestinal cells from scRNA-seq after oral PEDV infection, showing prominent activation of the RLR pathway. (**B**) Representative gross morphology of the small intestine from mock- and PEDV-infected piglets showing watery contents and intestinal distension after infection. (**C**) Histopathological analysis of jejunum and ileum from mock- and PEDV-infected piglets demonstrating villus atrophy and epithelial damage (H&E staining). Scale bars: 100 μm. (**D and E**) RT-qPCR quantification of RIG-I (**D**) and MDA5 (**E**) mRNA expression in jejunum and ileum tissues from mock- and PEDV-infected piglets. (**F**) KEGG enrichment of differentially expressed genes in intestinal epithelial cell populations from scRNA-seq following oral PEDV infection, highlighting antiviral innate immune pathways including RLR signaling. (**G**) Heatmap showing transcriptional expression profiles of RLR-associated genes in epithelial cell subsets derived from scRNA-seq data sets. (**H**) Immunofluorescence staining of jejunum and ileum sections showing RIG-I (green), EPCAM (red), and PEDV antigen (yellow). Nuclei are counterstained with DAPI (blue). Scale bars: 100 μm (main image) and 20 μm (inset). Line-scan analysis confirms spatial co-localization of RIG-I with PEDV^+^ epithelial cells. Data represent mean ± SD from at least three biological replicates. Statistical significance was determined by one-way ANOVA (**P < 0.01, ***P < 0.001; ns, not significant).

To systematically characterize how the intestinal mucosa senses PEDV, we reanalyzed a publicly available single-cell RNA-sequencing data set generated from the jejunum of piglets orally infected with PEDV. PEDV infection led to significant enrichment of multiple pathways associated with viral invasion and mucosal immune responses, including glutathione metabolism, adherens junctions, and TNF signaling (Fig. 1A). Among these, the RIG-I–like receptor (RLR) pathway showed the most prominent enrichment, suggesting a potential role in the early detection of PEDV by the intestinal mucosa. To validate these observations, we established a piglet oral PEDV infection model. At 24 h post-infection, piglets exhibited typical intestinal lesions (Fig. 1B), and H&E staining revealed marked villus atrophy and epithelial shedding in both the jejunum and ileum (Fig. 1C). Immunofluorescence staining further confirmed abundant PEDV antigen localized within intestinal epithelial cells (Fig. S1A). Consistent with these findings, PEDV infection markedly increased RIG-I transcript levels in both the jejunum and ileum (approximately 2–3-fold), whereas MDA5 was downregulated in the jejunum and remained largely unchanged in the ileum (Fig. 1D and E), indicating selective activation of the RIG-I pathway in the intestinal mucosa. Cell subset–resolved analysis further demonstrated that RLR pathway enrichment occurred predominantly in intestinal epithelial cells (Fig. 1F), with only minimal activation in myeloid, T, and B cell populations (Fig. S1B through D), indicating that epithelial cells are the principal contributors to RLR signaling in the mucosa. PEDV infection also enhanced the expression of key downstream signaling molecules, including STAT1, STAT2, IRF7, and NF-κB1, together with coordinated upregulation of canonical interferon-stimulated genes (ISGs), such as ISG15, OASL, MX1, IFIT1–3, and RSAD2, consistent with broad activation of the epithelial antiviral program. Notably, transcript levels of RIG-I were elevated in epithelial cells following infection, whereas TLR3 remained largely unchanged (Fig. 1G). This expression pattern, in parallel with the induction of IFN and ISG modules, suggests that early interferon signaling triggered by PEDV infection may contribute to the enhanced transcription of RIG-I. Immunofluorescence staining further supported these findings: although RIG-I is primarily expressed in lamina propria myeloid cells under steady-state conditions (Fig. S1E), PEDV infection led to strong induction of RIG-I within epithelial cells of both the jejunum and ileum, where it colocalized with viral antigen signals (Fig. 1H). Together, these results demonstrate that PEDV infection is primarily sensed through epithelial RIG-I signaling, which orchestrates early interferon-dependent antiviral responses and underscores the central role of RIG-I in intestinal mucosal innate immunity.

### RIG-I serves as the key sensor mediating interferon responses to PEDV

**Fig 2:**
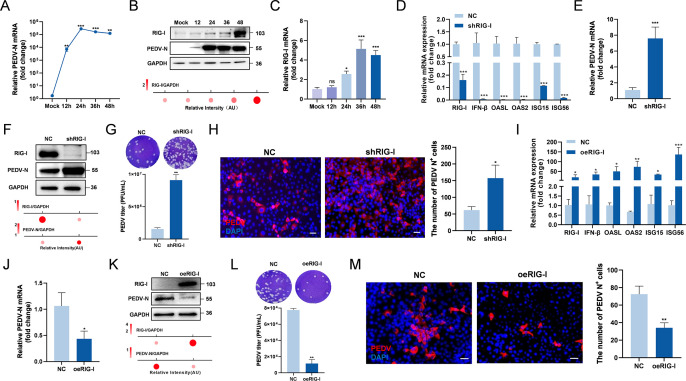
RIG-I functions as a major pattern recognition receptor sensing PEDV infection. (**A**) Time-course RT-qPCR analysis of PEDV N mRNA levels in MARC-145 cells at 12, 24, 36, and 48 h post-PEDV infection (hpi). (**B**) Western blot analysis of RIG-I protein expression in PEDV-infected MARC-145 cells at the indicated time points, with bubble plots summarizing quantitative band intensities. (**C**)Time-course RT-qPCR analysis of RIG-I mRNA levels in MARC-145 cells at 12, 24, 36, and 48 h post-PEDV infection (hpi). (**D and E**) RIG-I knockdown via shRNA in MARC-145 cells followed by PEDV infection. RT-qPCR analysis of RIG-I and downstream antiviral genes (**D**) and PEDV N RNA levels (**E**); Western blot evaluation of RIG-I and PEDV N proteins (**F**); plaque assays measuring extracellular viral titers (**G**); and immunofluorescence quantification of PEDV N-positive cells (**H**). (**I and J**) RT-qPCR analysis of RIG-I and downstream antiviral genes (IFN-β, OASL, OAS2, ISG15, ISG56) (**I**), as well as PEDV N RNA levels (**J**), in MARC-145 cells following RIG-I overexpression and PEDV infection. (**K**) Western blot confirming RIG-I overexpression and evaluating PEDV N protein abundance, with bubble plots showing densitometric quantification. (**L and M**) Viral replication in RIG-I–overexpressing cells assessed by plaque assays of culture supernatants (**L**) and immunofluorescence staining of PEDV N protein (red) with DAPI-labeled nuclei (blue) (**M**). Data represent mean ± SD from at least three independent experiments. Differences between the two groups were assessed by Student's *t*-test. For comparisons among three or more groups, statistical significance was determined by one-way ANOVA (*P < 0.05, **P < 0.01, ***P < 0.001; ns, not significant).

To investigate the contribution of RIG-I to PEDV sensing and antiviral signaling, we utilized MARC-145 cells, a well-established PEDV-susceptible cell line, to generate a robust *in vitro* infection model. PEDV demonstrated a rapid replication trajectory, with N-gene transcription peaking at 24 h and N-protein expression increasing further to its maximum at 48 h post-infection (Fig. 2A and B). The temporal kinetics of host responses paralleled viral replication, with both RIG-I mRNA and protein levels markedly upregulated at 24 h post-infection (Fig. 2B and C), whereas MDA5 exhibited a slight but non-significant decrease at both the transcript and protein levels (Fig. S2A and B).

To define the functional contribution of RIG-I, multiple shRNAs targeting RIG-I were designed and packaged into lentiviral vectors to establish stable shRNA-mediated epithelial cell lines with RIG-I knockdown (Fig. S2C and D). Among the generated lines, the shRIG-I-3 stable cell line exhibited more than 60% knockdown efficiency and was therefore used for subsequent experiments. Loss of RIG-I substantially suppressed the transcription of multiple ISGs, including OASL, OAS2, ISG15, ISG56, and IFN-β (Fig. 2D), while concomitantly enhancing PEDV replication, as evidenced by elevated N-gene transcription (Fig. 2E), increased N-protein abundance (Fig. 2F), and higher viral titers in the culture supernatant (Fig. 2G). In contrast, MDA5 knockdown exerted no measurable effect on viral replication (Fig. S2E and F). Immunofluorescence analysis corroborated these results, revealing a pronounced increase in red fluorescence intensity in RIG-I-deficient cells relative to negative controls, indicative of enhanced viral replication and spread (Fig. 2H). To validate these observations, we established complementary gain-of-function models by constructing stable RIG-I-overexpressing cell lines (Fig. S2G and H). Upon PEDV infection, cells overexpressing RIG-I exhibited robust activation of ISGs such as OASL and ISG15 (Fig. 2I), accompanied by marked reductions in viral N-gene transcription, N-protein expression, and viral titers (Fig. 2J and K). Immunofluorescence and plaque assays further confirmed a pronounced reduction in PEDV-specific red fluorescence and foci formation in RIG-I-overexpressing cells (Fig. 2L and M). Collectively, our data identify RIG-I as the dominant cytosolic sensor of PEDV and show that its downstream ISG activation is critical for restricting viral replication.

### RIG-I recognizes a functional PAMP in the PEDV 5′ ORF1a region

**Fig 3:**
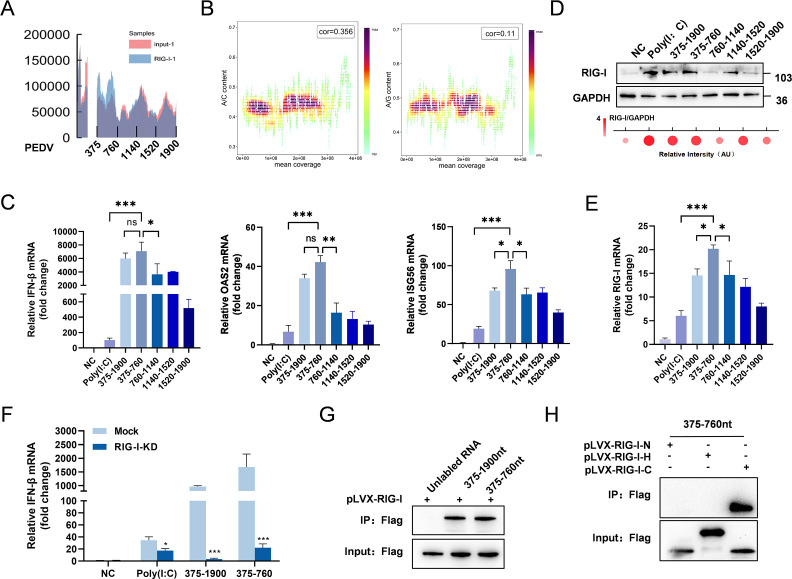
Identification and functional validation of PEDV RNA fragments interacting with RIG-I. (**A**) RIP-seq analysis identifying PEDV genomic regions enriched in RIG-I–associated RNA reads, with the strongest enrichment observed within the 5′ ORF1a region. (**B**) Nucleotide composition and motif analysis of enriched RIG-I binding regions, showing preferential enrichment of A/C- and A/G-rich sequence patterns. (**C–E**) Functional screening of truncated PEDV RNA fragments (375–760 nt, 760–1140 nt, 1140–1520 nt, and 1520–1900 nt) transfected into MARC-145 cells. (**C**) RT-qPCR analysis of IFN-β, OAS2, and ISG56 induction by each RNA fragment. (**D**) Western blot analysis of RIG-I protein expression, with quantitative densitometry shown below. (**E**) RT-qPCR quantification of RIG-I mRNA induction. (**F**) RT-qPCR evaluation of IFN-β mRNA induction in RIG-I–silenced MARC-145 cells following transfection with candidate RNA fragments, confirming RIG-I dependence of the observed responses. (**G and H**) RNA pull-down assays followed by Western blot validating the selective interaction between RIG-I and the 375–760 nt RNA fragment and mapping this interaction primarily to the CTD of RIG-I. “NC” represents the negative treatment of cells that only received the transfection reagent. For comparisons among three or more groups, statistical significance was determined by one-way ANOVA (*P < 0.05, **P < 0.01, ***P < 0.001; ns, not significant).

Given that RIG-I serves as the major cytosolic sensor of PEDV in the intestinal mucosa, we first performed RIP-seq to identify viral RNA regions directly bound by RIG-I. RIG-I binding was highly enriched within the 5′ portion of the ORF1a region (375–1900 nt), with a pronounced peak spanning nt 375-760 (Fig. 3A; Fig. S3A and Data S1). This region contains the viral gene initiation and ribosome entry sites, suggesting that it may harbor structural elements more accessible for host recognition. Motif-scanning analysis further revealed enrichment of A/C- and A/G-rich nucleotide patterns (Fig. 3B and Fig. S3B), indicating that specific sequence features may contribute to RIG-I recognition of PEDV RNA. To assess the functional importance of this enriched region, we generated a series of truncated RNA fragments covering nt 375–1900, including 375–760 nt, 760–1140 nt, 1140–1520 nt, and 1520–1900 nt. Among these, the 375–760 nt fragment exhibited the strongest immunostimulatory activity, robustly inducing IFN-β transcription and secretion and coordinately upregulating multiple downstream antiviral genes (Fig. 3C and Fig. S3C). Its stimulatory capacity was comparable to the full-length 375–1900 nt fragment and markedly exceeded that of Poly(I:C) (Fig. S3D), identifying 375–760 nt as one of the most potent RIG-I–activating regions within the PEDV genome.

Further analysis showed that the 375–760 nt fragment significantly increased RIG-I transcription and protein expression (Fig. 3D and E). However, this fragment failed to induce interferon responses in RIG-I–knockdown cells (Fig. 3F) and did not significantly alter MDA5 mRNA expression in control cells (Fig. S3E). These results support a preferential requirement for RIG-I in sensing the 375–760 nt PEDV PAMP under our experimental conditions. Finally, RNA pull-down assays revealed a direct and specific interaction between the 375–760 nt RNA and RIG-I. Binding required the C-terminal domain (CTD) of RIG-I, whereas the N-terminal CARDs and helicase domain displayed little to no binding (Fig. 3G and H), indicating that the CTD mediates recognition of this structured RNA element. Together, these findings demonstrate that the 375–760 nt region within the 5′ ORF1a segment of the PEDV genome represents a structurally defined and functionally dominant PAMP, which is directly recognized by the RIG-I CTD and potently activates type I interferon signaling. Similarly, we conducted key validation experiments for PEDV PAMPs using the porcine intestinal organoid system. The results showed trends consistent with those observed in *in vitro* experiments using MARC-145 cells (Fig. S4A through F). These results support the ability of the 375–760 nt PEDV PAMP to activate RIG-I–IFN signaling and restrict PEDV replication in a porcine intestinal epithelial model.

To assess the conservation of the identified PEDV PAMP region, we analyzed complete PEDV genome sequences representing different genotypes together with the ZJ08 strain used in this study. The 375–760 nt region was extracted based on the ZJ08 genomic coordinates and aligned using MAFFT, followed by manual inspection in MEGA. WebLogo visualization revealed a dominant nucleotide at most positions, with only limited polymorphic sites across the analyzed strains (Fig. S5). These findings indicate that the 375–760 nt region is overall conserved among representative PEDV strains, supporting its potential relevance as a broadly conserved immunostimulatory RNA element.

### Molecular basis of RIG-I binding and activation by PEDV-derived PAMP

**Fig 4:**
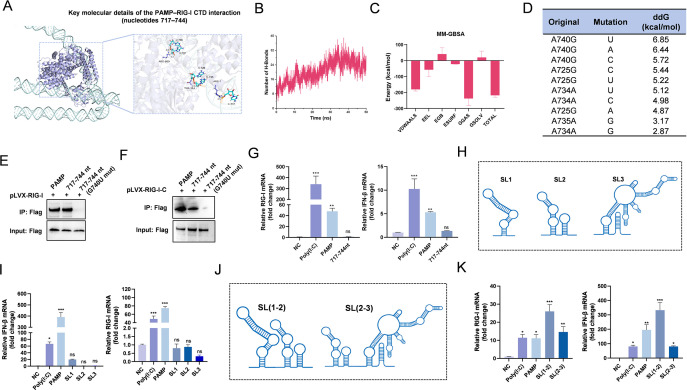
Structural characterization of the PEDV-derived PAMP and mapping of its RIG-I interaction interface. (**A**) Molecular docking between RIG-I and the 375–760 nt PEDV RNA fragment. (**B and C**) Molecular dynamics simulations depicting the interaction between RIG-I and the 717–744 nt PEDV RNA fragment, illustrating predicted binding conformations and interface stability. The number of hydrogen bonds was analyzed to investigate the variation of the number of hydrogen bonds formed between nucleic acids and proteins over simulation time (**B**). Combined with free energy analysis, the movement trajectories of protein-nucleic acid complexes in the 40–50 ns time period were extracted to break down the energy term (**C**). (**D–F**) Site-directed mutagenesis combined with RNA pull-down assays identifying critical nucleotides mediating RNA–RIG-I interaction and confirming the predominant involvement of the RIG-I CTD in recognizing the viral RNA fragment. (**G**) Functional assessment of the 717–744 nt sub-fragment in MARC-145 cells compared with Poly(I:C) and the full-length PAMP, demonstrating its inability to induce RIG-I-dependent IFN responses. (**H–K**) Mfold-based predictions of RNA secondary structure and functional evaluation of individual and combined stem-loop elements (SL1, SL2, SL3, SL1–2, SL2-3, and composite constructs). RT-qPCR analysis reveals which stem-loop architectures are required for optimal RIG-I activation. “NC” represents the negative treatment of cells that only received the transfection reagent. Data represent mean ± SD from at least three independent experiments. Statistical significance was determined by one-way ANOVA (*P < 0.05, **P < 0.01, ***P < 0.001; ns, not significant).

To elucidate the structural basis underlying RIG-I recognition of PEDV RNA, molecular docking and molecular dynamics (MD) simulations were performed using the 375–760 nt PAMP fragment. Rosetta-based free energy analysis revealed a binding energy of –32.7 kcal/mol, suggesting a strong interaction between RIG-I and the PAMP RNA. Docking analysis indicated that the PAMP binds stably to the CTD of RIG-I, where the 717–744 nt region forms multiple hydrogen bonds and salt bridges with key residues, including ARG-7, THR-354, and ARG-869. These residues are distributed across different regions of the binding pocket, forming a multipoint recognition network that stabilizes the complex. Specifically, ARG-7 forms a 4.5 Å salt bridge with C-717, while THR-354 forms a 2.9 Å hydrogen bond with G-725/G-726, thereby anchoring the RNA backbone (Fig. 4A).

MD simulations demonstrated the structural stability of the RIG-I–RNA complex, with RMSD values showing minimal fluctuations within the first 10 ns and stabilizing at ~1.3 nm after 40 ns (Fig. S6A). Hydrogen bond analysis revealed a gradual increase in hydrogen bond number during the early phase of simulation, stabilizing between 20 and 28 bonds after 35 ns (Fig. 4B). Binding free energy decomposition showed that van der Waals interactions (–180.24 kcal/mol) were the primary driving force, with electrostatic interactions (–56.97 kcal/mol) contributing significantly to complex stabilization (Fig. 4C). Further energetic analysis identified several nucleotides within the 717–744 nt region—G725, G726, A734, A735, and G740—as key contributors to binding affinity and complex stability (Fig. S6B). Virtual saturation mutagenesis revealed that substitution of G740 with U resulted in the largest increase in binding free energy, indicating that this nucleotide plays a critical role in stabilizing the RIG-I–RNA complex (Fig. 4D).

RNA pull-down assays confirmed direct binding of the 717–744 nt fragment to both full-length RIG-I and its CTD, while the G740 mutation effectively abolished this interaction (Fig. 4E and F). Interestingly, despite stable binding, the 717–744 nt fragment failed to induce IFN production (Fig. 4G), suggesting that binding alone is insufficient for RIG-I activation and that conformational features of RNA may be required for downstream signaling. Secondary structure prediction of the 375–760 nt region revealed three conserved stem-loop structures (Fig. 4H). Functional analyses showed that individual stem-loops (SL1, SL2, or SL3) were insufficient to activate RIG-I or induce appreciable IFN-β expression (Fig. 4I). In contrast, dual stem-loop constructs (SL1–2 or SL2–3) markedly enhanced RIG-I transcription by 10–20-fold and IFN-β expression by 100–200-fold (Fig. 4J and K). Moreover, co-transfection of distinct single stem-loops partially recapitulated this synergistic effect (Fig. S6C), further supporting the requirement for cooperative interactions among multiple stem-loop structures in driving effective RIG-I activation. The stem-loops did not significantly alter the expression of MDA5 mRNA in the cells (Fig. S3E). Together, these findings demonstrate that RIG-I activation requires not only RNA binding but also cooperative presentation of multiple stem-loop structures, which enables effective downstream signal transduction.

### PEDV-derived PAMP activates innate immunity and confers broad antiviral protection

**Fig 5:**
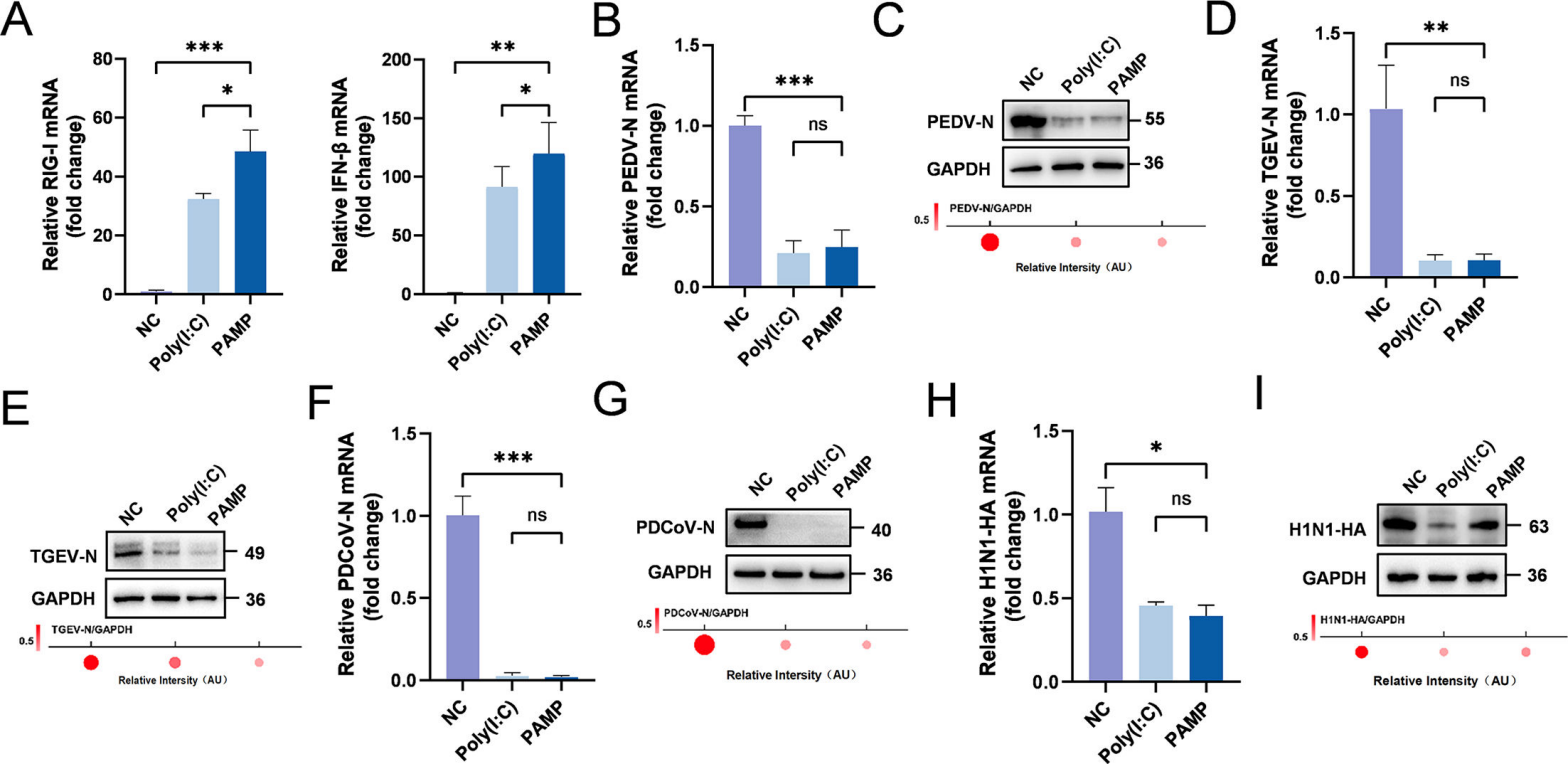
PEDV-derived PAMP RNA activates RIG-I signaling and confers broad-spectrum antiviral protection. (**A–C**) MARC-145 cells were transfected with control RNA (NC), Poly(I:C), or PAMP RNA at 6 h prior to PEDV infection. RT-qPCR analysis of RIG-I and IFN-β mRNA expression (**A**), PEDV N RNA levels (**B**), and Western blot quantification of PEDV N protein (**C**) at 24 h post-infection. (**D and E**) RT-qPCR quantification of TGEV N mRNA (**D**) and Western blot analysis of TGEV N protein (**E**) in ST cells pre-treated with NC, Poly(I:C), or PAMP RNA and then infected with TGEV. (**F and G**) RT-qPCR analysis of PDCoV N mRNA (**F**) and Western blot quantification of PDCoV N protein (**G**) in ST cells following PAMP or Poly(I:C) transfection and PDCoV infection. (**H and I**) RT-qPCR measurement of H1N1 HA mRNA (**H**) and Western blot analysis of HA protein expression (**I**) in human A549 cells transfected with NC, Poly(I:C), or PAMP RNA before H1N1 infection. Data represent mean ± SD from at least three independent experiments. Statistical significance was determined by one-way ANOVA (*P < 0.05, **P < 0.01, ***P < 0.001; ns, not significant).

After elucidating the molecular basis of RIG-I recognition of the PEDV PAMP, we next evaluated its capacity to activate downstream interferon signaling and its antiviral efficacy. Poly(I:C), a classical double-stranded RNA analog that stimulates interferon production through RIG-I/MDA5–dependent pathways, served as a positive control. In PEDV-infected MARC-145 epithelial cells, transfection with the PEDV PAMP markedly upregulated RIG-I and IFN-β transcription (Fig. 5A). Both PEDV N-gene transcription and N-protein expression decreased by approximately 70% following PAMP stimulation (Fig. 5B and C), indicating that PEDV PAMP restricts viral replication through robust activation of the RIG-I–IFN-β axis. Notably, PEDV PAMP induced much stronger RIG-I transcription than Poly(I:C), suggesting higher intrinsic immunostimulatory potency. We further investigated whether the G740 site within the PAMP, which affects RIG-I binding, influences viral replication. The results showed that, compared with wild-type RNA, the RNA with the G740 mutation had no significant effect on PEDV replication (Fig. S6D), indicating that G740 still specifically affects RIG-I binding without sufficient impact on RIG-I function.

Given the central role of RIG-I signaling in antiviral defense, we next investigated whether the PEDV PAMP could exert antiviral activity beyond the PEDV system. In porcine ST epithelial cells infected with TGEV or PDCoV, PAMP transfection consistently activated the RIG-I pathway and strongly induced IFN-β transcription (Fig. S7A and B). Correspondingly, PEDV PAMP suppressed TGEV and PDCoV replication in ST cells by over 90%, as demonstrated by marked reductions in viral N-gene transcripts and protein levels (Fig. 5D through G). We also examined the activity of the PEDV-derived PAMP in an H1N1 infection system. In A549 cells, PAMP stimulation reduced H1N1 HA mRNA and protein expression to ~50% of control levels (Fig. 5H and I), supporting the broader innate immune–activating potential of this RNA element.

We next evaluated the immunostimulatory activity of the PEDV-derived PAMP *in vivo*. Mice were administered the PAMP via either intravenous (IV) or intramuscular (IM) injection, whereas piglets received the PAMP intramuscularly (Fig. S8A). Serum and tissue samples were collected 24 h after administration to assess innate immune activation. In mice, both IV and IM delivery of the PAMP rapidly elicited systemic innate immune responses. Serum cytokine measurements revealed significantly elevated IFN-β and TNF-α levels relative to NC and Poly(I:C) controls, with IM injection inducing the strongest response (Fig. S8B). Heatmap analysis further demonstrated robust upregulation of RIG-I, IFN-β, and multiple ISGs—including OASL, OAS2, ISG15, and ISG56—in the spleen and lung, again with IM administration producing the greatest transcriptional activation (Fig. S8C). These findings establish that the PEDV PAMP acts as a potent systemic innate immune stimulant *in vivo*. A similar pattern of innate immune activation was observed in piglets. Intramuscular administration of the PAMP markedly increased serum IFN-β levels within 24 h (Fig. S8D), indicating rapid systemic antiviral cytokine induction in the natural host. Concurrently, the jejunum and ileum exhibited pronounced transcriptional upregulation of RIG-I and IFN-β (Fig. S8E), demonstrating that the PAMP effectively engages antiviral signaling at the intestinal mucosal surface in addition to systemic circulation. Together, these results show that the PEDV-derived PAMP activates both systemic and mucosal innate immune pathways *in vivo*, supporting its potential as a natural immunostimulant for enhancing antiviral defenses in swine.

**Fig 6:**
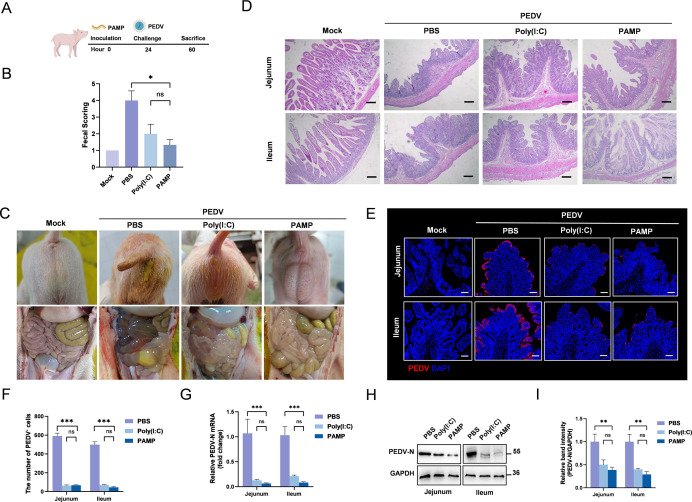
PEDV-derived PAMP RNA enhances antiviral innate immunity and protects piglets from PEDV challenge. (**A**) Experimental design of the piglet infection model comprising four treatment groups: mock group, orally administered culture medium as a mock challenge and intramuscularly injected with PBS; PBS group, orally challenged with PEDV and intramuscularly injected with PBS; Poly(I:C) group, orally challenged with PEDV and intramuscularly administered Poly(I:C); and PAMP group, orally challenged with PEDV and intramuscularly administered PAMP (*n* = 3 per group). (**B and C**) Fecal scoring (**B**), together with clinical manifestations and gross pathological examination of small intestine tissues (**C**), shows attenuation of diarrhea and mucosal damage in PAMP-treated piglets compared with controls. (**D–F**) Histological and immunofluorescence analyses of intestinal tissues. Hematoxylin and eosin (H&E) staining of small intestinal sections (**D**). Immunofluorescence staining detecting PEDV antigen (**E**) and quantification of PEDV-positive epithelial cells (**F**). (**G–I**) Viral load assessment in intestinal tissues. RT-qPCR analysis of PEDV N RNA levels in the jejunum and ileum (**G**), and Western blot analysis of viral N protein expression (**H**), along with quantitative analysis of protein levels (**I**), collectively demonstrating reduced viral replication in PAMP-treated piglets. Data represent mean ± SD from at least three independent experiments. Statistical significance was determined by one-way ANOVA (*P < 0.05, **P < 0.01, ***P < 0.001; ns, not significant).

In the PEDV challenge model, this PAMP-induced innate immunity conferred pronounced clinical protection (Fig. 6A). Fecal scoring revealed stark differences across treatment groups (Fig. 6B). Piglets in the PBS-treated group rapidly developed severe watery diarrhea (score ~4), whereas Poly(I:C)- and PAMP-treated piglets maintained scores of ~2 (mild soft stool) and 1–2 (normal to mildly soft stool), respectively, with no evidence of watery diarrhea. PBS-treated piglets exhibited extensive perianal soiling, watery intestinal contents, and marked intestinal distension, whereas PAMP- and Poly(I:C)-treated piglets showed largely normal intestinal morphology (Fig. 6C). Histological and immunofluorescence analyses further confirmed the protective effects (Fig. 6D through F). PBS-treated piglets showed severe villus atrophy, epithelial disruption, and abundant PEDV antigen in the jejunum and ileum, whereas these abnormalities were markedly reduced in the PAMP and Poly(I:C) groups. Virological assays were consistent with these findings, showing reduced PEDV N RNA and N protein levels in both intestinal segments (Fig. 6G through I). Together, these results demonstrate that PEDV PAMP potently activates interferon-driven innate immunity, restricts viral replication and dissemination, preserves intestinal structural integrity, and provides protective efficacy in the natural host comparable to Poly(I:C).

### PEDV-derived PAMP functions as a potent RIG-I agonist to enhance inactivated vaccine-induced immune protection

**Fig 7:**
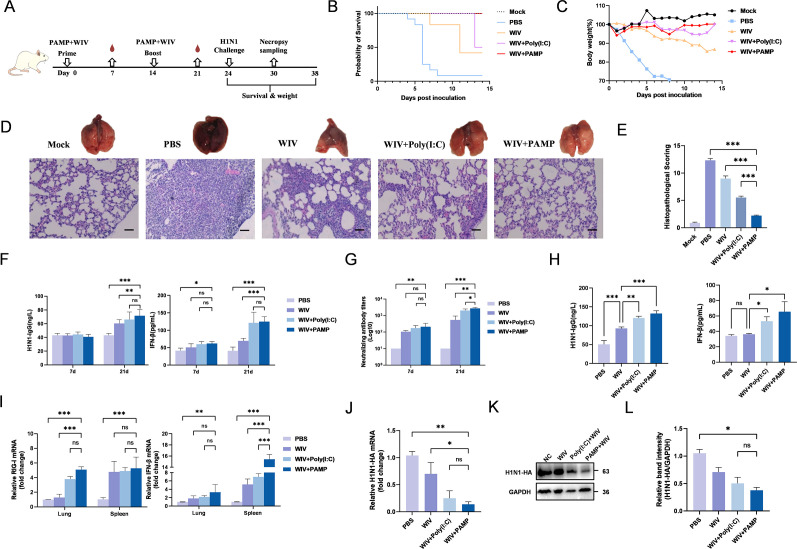
PEDV-derived PAMP RNA enhances innate immune activation and potentiates vaccine-mediated protection. (**A**) Immunization and viral challenge schedule in BALB/c mice, including five groups (*n* = 10 per group): mock group: intramuscular PBS, mock WIV immunization (culture medium), and mock H1N1 challenge (PBS); PBS group: intramuscular PBS, mock WIV immunization (culture medum), and H1N1 challenge; WIV group: intramuscular PBS, WIV immunization, and H1N1 challenge; WIV + Poly(I:C) group: intramuscular Poly(I:C), WIV immunization, and H1N1 challenge; WIV + PAMP group: intramuscular PAMP, WIV immunization, and H1N1 challenge. (**B and C**) Survival curves (**B**) and body weight changes (**C**) of mice following influenza virus challenge, showing enhanced protection in the WIV + PAMP group. (**D and E**) Histopathological examination of lung tissues (**D**) with corresponding pathology scores (**E**), revealing reduced inflammation and tissue damage in WIV + PAMP–immunized mice. (**F–H**) Humoral and innate immune responses. Serum IFN-β and IgG levels measured at days 7 and 21 post-vaccination (**F**), neutralizing antibody titers (**G**), and IFN-β and IgG levels in bronchoalveolar lavage fluid at day 6 post-infection (**H**). PAMP co-administration markedly enhances systemic and mucosal immune responses. (**I–L**) Lung innate immunity and viral load assessment. RT-qPCR quantification of RIG-I and IFN-β mRNA (**I**), RT-qPCR measurement of viral HA mRNA (**J**), and Western blot detection of HA protein in lung homogenates (**K**), along with protein statistical graphs (**L**), confirming reduced viral replication in WIV + PAMP–vaccinated mice. Data represent mean ± SD from at least three independent experiments. Statistical significance was determined by one-way ANOVA (*P < 0.05, **P < 0.01, ***P < 0.001; ns, not significant).

Previous findings demonstrated that intramuscular administration of PEDV PAMP activates robust RIG-I-mediated interferon signaling in both the intestine and the lung, thereby enhancing innate antiviral defense. Because RIG-I-driven type I interferon responses also play an essential role in facilitating antigen presentation and T-cell activation (25), we hypothesized that the PEDV PAMP may not only possess broad-spectrum antiviral activity but could also potentiate vaccine-induced adaptive immunity through RIG-I–dependent pathways. To test this hypothesis, we established a mouse immunization model using a whole inactivated influenza virus (WIV) vaccine (Fig. 7A). Compared with mice receiving WIV alone, those immunized with the WIV + PAMP combination displayed markedly improved protection after viral challenge. PBS mice began to succumb on day 4 post-infection, and only one animal survived by day 8, accompanied by substantial weight loss, lethargy, and respiratory distress. WIV alone provided partial protection, but vaccinated mice still exhibited ~15% body weight loss and ~30% mortality. Strikingly, all mice in the WIV + PAMP group survived the challenge, maintained stable body weight, and showed no clinical symptoms throughout the observation period (Fig. 7B and C). Histopathological examination further confirmed these enhanced protective effects. PBS mice showed diffuse pulmonary hemorrhage, alveolar collapse, and severe inflammatory infiltration. WIV alone mitigated pathology to some extent but still resulted in focal inflammation and mild interstitial damage. In contrast, lungs from WIV + PAMP-immunized mice exhibited near-normal architecture, with only minimal congestion and sparse mononuclear infiltration (Fig. 7D and E), indicating that PEDV PAMP markedly attenuates virus-induced lung injury. Serological analyses supported these findings. Compared with WIV alone, the WIV + PAMP regimen induced an approximately 3.2-fold increase in serum IFN-β levels at day 21 post-immunization (Fig. 7F). Total IgG levels increased by approximately 2.5-fold, and neutralizing antibody titers were elevated by approximately 2 logs (Fig. 7F and G), demonstrating that PAMP co-administration drives a stronger and more durable humoral response. To determine whether mucosal immunity was similarly enhanced, we measured IFN-β and IgG levels in bronchoalveolar lavage fluid (BALF) on day 6 post-challenge. Both readouts were significantly higher in the WIV + PAMP group compared with all other groups (Fig. 7H), consistent with the systemic serological findings and indicating that PEDV PAMP concurrently strengthens both systemic and respiratory mucosal immune responses. At the molecular level, co-immunization with WIV + PAMP increased lung RIG-I and IFN-β transcript levels by 2–3-fold relative to WIV alone (Fig. 7I). Correspondingly, influenza viral HA mRNA and protein abundance decreased by more than 70% (Fig. 7J through L), confirming strong suppression of viral replication. Collectively, these findings demonstrate that PEDV PAMP functions as a potent RIG-I Poly(I:C)agonist that amplifies interferon-driven innate immunity and substantially enhances antigen-specific adaptive responses elicited by inactivated vaccines.

## DISCUSSION

Innate immune recognition through PRRs constitutes the first line of defense against viral infection. Although numerous studies have implicated various PRRs in sensing RNA viruses and triggering interferon responses (26, 27), most of these findings are based on simplified *in vitro* systems that fail to recapitulate the cellular and spatial complexity of mucosal infection. By integrating single-cell transcriptomic profiling with both *in vivo* and *in vitro* infection models, we identified RIG-I, rather than MDA5, as the predominant RLR sensor mediating antiviral recognition in the intestinal mucosa during PEDV infection. Under homeostatic conditions, RIG-I is expressed at very low levels in epithelial cells; however, upon viral entry, it is rapidly induced, leading to robust activation of interferon signaling and efficient suppression of viral replication. This inducible expression pattern aligns with previously reported RIG-I regulatory mechanisms, collectively suggesting that RIG-I achieves a dynamic balance between immune vigilance and tolerance (28). Such a strategy prevents inappropriate activation by commensal or non-pathogenic RNAs under steady-state conditions while enabling rapid deployment of mucosal antiviral defenses upon infection, thereby providing a temporal advantage for the initiation of adaptive immunity (29). Notably, MDA5 expression remained largely unchanged in PEDV-infected epithelial cells, and MDA5 silencing did not measurably affect viral replication. These results indicate that MDA5 likely functions as a bystander in the global antiviral response rather than as a direct viral sensor. Based on viral RNA feature analysis, we propose that the functional divergence between RIG-I and MDA5 primarily arises from ligand specificity. Previous studies have demonstrated that MDA5 preferentially recognizes long dsRNA, whereas PEDV replication predominantly generates short to intermediate dsRNA fragments that are more efficiently detected by RIG-I (11, 30). Consequently, during PEDV infection, RIG-I acts as the principal sensor that triggers viral recognition and amplifies interferon signaling, while MDA5 plays a secondary, supportive role in the broader antiviral activation. Collectively, our findings establish RIG-I as the central driver of mucosal antiviral defense during PEDV infection and reveal a context-dependent functional hierarchy within the RLR family, shaped by both the tissue environment and ligand characteristics. These insights advance our understanding of coronavirus sensing at mucosal surfaces and provide a conceptual framework for deciphering innate immune recognition in enteric viral infections.

Using RIP-seq, we mapped the interaction landscape between RIG-I and the PEDV genome and identified binding sites predominantly enriched at the 5′ end of the positive-sense ORF1a region. This region harbors essential elements for replication and translation initiation and generates abundant RNA intermediates early during infection (31), suggesting that it serves as the major ligand for RIG-I recognition. Motif analysis further revealed a preference for A/C- and A/G-rich sequences, consistent with RIG-I’s intrinsic bias toward adenosine- and guanosine-rich motifs (32). Such sequence selectivity sharpens discrimination between viral and host RNAs and stabilizes recognition of conserved structural features, enabling RIG-I to maintain robust antiviral surveillance despite viral sequence variation. Comparison with other RNA viruses underscores the diversity of RLR–virus interactions. Whereas PRRSV is sensed through its 3′UTR, influenza virus exposes panhandle structures, and flaviviruses rely on highly structured 5′ termini (18, 19, 33), PEDV engages RIG-I through early exposure of its ORF1a transcripts, reflecting divergent replication strategies and immune evasion mechanisms among RNA viruses. Notably, despite effective sensing at the onset of replication, PEDV still establishes robust mucosal infection by deploying multiple immune antagonists: nsp1 blocks IRF3 activation, nsp15 degrades dsRNA to limit RIG-I ligands, and ORF3 suppresses interferon production (34, 35). In this context, early immune activation paradoxically drives excessive inflammation that compromises mucosal integrity, facilitating viral replication and persistence and ultimately exacerbating intestinal pathology.

To clarify how PEDV RNAs are recognized by RIG-I and what structural features govern their activation, we combined RNA pull-down assays with structural–functional analyses. We identified nucleotides 375–760 within the ORF1a region as a critical PAMP that directly binds the CTD of RIG-I to initiate innate immune signaling. Docking and pull-down experiments further pinpointed a ~30 nt fragment that forms stable hydrogen bond and hydrophobic contacts with RIG-I but fails to trigger interferon production, indicating that RNA binding alone is necessary but insufficient to relieve RIG-I autoinhibition. Previous studies have demonstrated that efficient RIG-I activation requires not only canonical features such as 5′ppp and RNA duplexes but also higher-order RNA conformations capable of disrupting its autoinhibited state (36). Consistent with this principle, our findings reveal that PEDV PAMP activity is conformation-dependent: single stem-loops were ineffective, whereas double or multiple stem-loop assemblies strongly enhanced RIG-I activation, reaching levels comparable to the full-length PAMP. This conformational requirement parallels activation modes observed in other RNA viruses, such as PRRSV, where RIG-I activation depends on cooperation between a 3′UTR pseudoknot and adjacent dsRNA (33), or HCV, which requires the poly-U/UC tract paired with a short duplex RNA (37). Together, these results define PEDV PAMPs as conformation-dependent activators of RIG-I, revealing an evolutionary strategy in which RNA viruses sculpt complex RNA architectures to balance immune recognition and evasion. This mechanism enables viruses to retain essential replication elements while minimizing premature immune clearance. Moreover, our study highlights that the potency of RNA-based immunostimulants depends not only on molecular signatures such as 5′ppp but also on higher-order RNA structures, suggesting that rational incorporation of stem-loops or pseudoknots could greatly enhance their immunostimulatory efficacy.

Conventional adjuvants have greatly advanced vaccine development but continue to present critical limitations. Classical TLR-targeting adjuvants, such as LPS (TLR4 ligand), elicit strong immune activation yet display prohibitive toxicity, whereas its derivative MPLA achieves better safety at the cost of reduced potency (38). Likewise, CpG ODN (TLR9 ligand) enhances humoral immunity but provides only modest stimulation of cellular responses and depends heavily on the host immune milieu (39). These shortcomings highlight the need for next-generation adjuvants that combine potency with safety. In this study, the PEDV-derived PAMP we identified exhibits distinct immunostimulatory properties. Acting as a viral RIG-I ligand, it mimics natural infection by activating the RIG-I–MAVS axis, inducing robust interferon and ISG expression, and establishing a durable intracellular antiviral state. Functionally, this RNA motif not only inhibited PEDV and related porcine enteric coronaviruses but also showed additional antiviral activity in an H1N1 infection system, supporting the broader innate immune–activating potential of this PEDV-derived PAMP. The concept of exploiting viral RNAs as innate immune stimulants has been substantiated previously: Sendai virus 5′ppp-RNA induces broad-spectrum activity against several enteric and respiratory RNA viruses (40), and the poly-U/UC tract of hepatitis C virus serves as a potent RIG-I agonist that boosts vaccine efficacy (41). Together, these findings position the PEDV RNA recognized by RIG-I as a promising natural immunoadjuvant capable of reinforcing protective immunity. Building upon these observations, our data also raise the possibility that the PEDV-derived PAMP may exert adjuvant effects that extend beyond the enhancement of humoral immunity. Given the central role of RIG-I signaling in promoting dendritic-cell maturation, type I interferon production, and cytotoxic T-cell priming, activation of this pathway by the PEDV PAMP may facilitate more efficient antigen presentation and potentiate Th1-skewed cellular immune responses. Although our current study primarily assessed antibody-mediated protection, future investigations examining antigen-specific CD4^+^ and CD8^+^ T-cell responses will be essential for determining whether this PAMP can function as a dual-acting adjuvant capable of strengthening both humoral and cellular immunity, thereby expanding its translational potential in vaccine development. Given that RIG-I agonists can enhance dendritic-cell cross-presentation and upregulate co-stimulatory molecules—thereby facilitating antigen processing through the MHC I pathway and promoting CD8^+^ T-cell expansion and effector differentiation (42)—the PEDV PAMP is likely to elicit both strong innate activation and Th1-skewed adaptive responses while potentiating cytotoxic T-lymphocyte activity. Such dual activation is particularly advantageous for vaccines targeting intracellular pathogens or highly variable viruses. Future studies should systematically assess the *in vivo* adjuvant efficacy and safety of PEDV PAMP, explore its applicability across different vaccine platforms, and refine its delivery strategies to fully realize its immunostimulatory potential, thereby laying the foundation for its development as a next-generation adjuvant with both high safety and strong immunogenicity.

**Fig 8:**
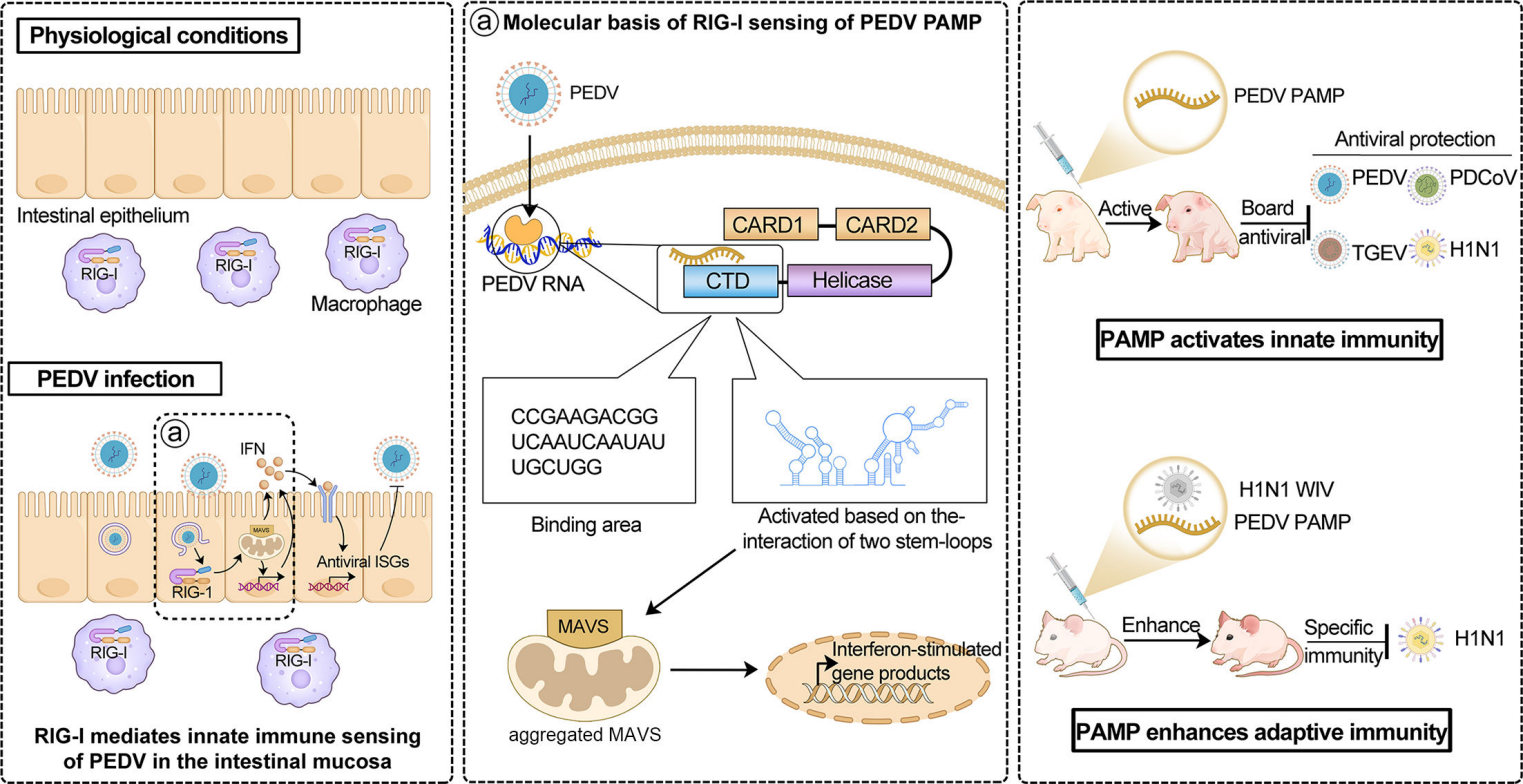
Mechanistic model of RIG-I recognition and innate immune activation by a structurally defined PEDV PAMP.

Through integration of intestinal single-cell transcriptomics with *in vitro* and *in vivo* infection models, we demonstrate that RIG-I serves as a critical determinant of mucosal defense against PEDV. Under physiological homeostasis, RIG-I in the intestinal mucosa is predominantly expressed by mononuclear cells, with minimal expression in epithelial cells; however, it is rapidly upregulated in epithelial cells upon viral infection, thereby activating downstream interferon pathways and exerting antiviral effects. RIP-seq analysis further identified the 5′ ORF1a region of the PEDV genome as the primary RIG-I target, defined by specific nucleotide preferences and stable secondary structures that constitute its molecular basis as a critical PAMP. Importantly, RIG-I activation proved to be conformation-dependent: single stem-loops were insufficient to trigger signaling, whereas double or multiple stem-loop assemblies markedly enhanced immune responses, revealing that effective activation requires higher-order RNA architectures. Functionally, the identified PEDV PAMP not only restricted replication of multiple RNA viruses through the RIG-I–MAVS axis but also augmented both innate and adaptive mucosal immunity, underscoring its potential as a novel immunoadjuvant (Fig. 8). Collectively, these findings deepen our understanding of how coronaviruses are sensed at mucosal surfaces and provide a conceptual framework for harnessing PRR pathways in antiviral target discovery and the rational design of next-generation mucosal vaccines.

## MATERIALS AND METHODS

### Reagents and antibodies

High-glucose Dulbecco’s modified Eagle’s medium (DMEM), fetal bovine serum (FBS), and 0.25% trypsin (SenBeiJia, Nanjing, China); TRIzol reagent, cDNA synthesis, and high-fidelity DNA polymerase (Vazyme, Nanjing, China); DNA gel recovery and plasmid extraction kits (Omega Bio-Tek, USA); RIPA lysis buffer and PMSF (Biosharp, Hefei, China); and protein markers (GenScript, Nanjing, China) were used for molecular and biochemical assays. Lipofectamine 3000 (Thermo Fisher Scientific, USA) was used for transfection. For viral infection assays, puromycin (2 μg/mL; Sigma-Aldrich, USA) was used for selection and polybrene (8 μg/mL; Sigma) to enhance transduction. Poly(I:C), used as a positive stimulator for RIG-I activation, was purchased from Beyotime (Shanghai, China). ELISA kits for monkey and mouse IFN-β and mouse TNF-α were obtained from AiFang Biotech (Hunan, China). All other reagents and chemicals were purchased from Vazyme unless otherwise specified.

Monoclonal antibodies against the nucleocapsid (N) proteins of TGEV, PDCoV, and PEDV were used in this study. The anti-TGEV N protein antibody was generated in our laboratory. The anti-PEDV N monoclonal antibody was custom-made by Jinsirui Biotechnology Co., Ltd. (Nanjing, China) and stored in our laboratory (diluted 1:200 for immunofluorescence and 1:1,000 for Western blot analysis). The anti-PDCoV N monoclonal antibody was purchased from Medgene Labs (USA). A monoclonal antibody against RIG-I (Cell Signaling Technology, USA; 1:1,000 for WB) was also used. For epitope detection, mouse anti-Flag (Beyotime, Shanghai, China; 1:1,000 for WB) and anti-H1N1-HA (Sino Biological, Beijing, China; 1:200 for IF, 1:1,000 for WB) monoclonal antibodies were employed. Secondary antibodies included HRP-conjugated goat anti-mouse IgG and HRP-conjugated goat anti-rabbit IgG (Yeasen Biotechnology, Shanghai, China; 1:10,000 for WB).

### Cells and viruses

MARC-145 (a monkey kidney–derived cell line), Vero E6, ST (swine testis), and A549 cell lines were maintained in our laboratory, whereas HEK293T cells were obtained from the American Type Culture Collection (ATCC). All cell lines were cultured in high-glucose DMEM, supplemented with 10% FBS and incubated at 37°C in a humidified atmosphere containing 5% CO₂. The PEDV ZJ08 (GenBank: JX002693), TGEV SHXB (GenBank: KP202848), and influenza virus H1N1 A/PR/8/34 (GenBank: ACV49549.1) were maintained in our laboratory, and the PDCoV CH/JX/JGS/01 strain (GenBank: KY293677) was kindly provided by Yuxin Tang (Jiangxi Agricultural University). PEDV was propagated in Vero E6 cells, and TGEV and PDCoV were propagated in ST cells; H1N1 was amplified in A549 cells. Confluent monolayers were infected at 37°C for 1 h, washed to remove unbound virus, and maintained in DMEM containing 1% FBS. Viral stocks were collected after cytopathic effect development, clarified following a freeze–thaw cycle, titrated by plaque assay, and stored at −80℃. All cell lines were routinely confirmed to be mycoplasma-free.

### Porcine intestinal organoid culture and organoid-derived epithelial monolayer assays

Porcine small intestinal organoids were established from jejunal crypts isolated from PEDV-negative neonatal piglets as described previously with minor modifications (43). Crypts were embedded in growth factor–reduced Matrigel (Corning, Cat. No. 356231) and cultured in complete porcine intestinal organoid medium (OGM), as described previously, containing essential niche factors, including Wnt3a, R-spondin 1, Noggin, EGF, N-acetylcysteine, B27 (minus vitamin A), and N2 supplement, with the addition of Y-27632 dihydrochloride (10 μM) and Primocin (100 μg/mL) during initial culture. Organoids were maintained at 37°C in 5% CO₂, passaged every 5 days, and used for experiments between passages 3 and 6. For two-dimensional epithelial monolayer culture, 3D organoids were dissociated into small fragments using TrypLE Express (Thermo Fisher Scientific) and seeded onto Matrigel-coated 24-well plates at a density of approximately 1 × 10⁵ cells per well. Monolayers were cultured until reaching approximately 80% confluence before infection or RNA transfection. For PEDV infection assays, organoid-derived monolayers were infected with PEDV ZJ08 at an MOI of 0.1 for 48 h. For PAMP stimulation assays, monolayers were transfected with Poly(I:C) or *in vitro*-transcribed 375–760 nt PEDV PAMP RNA using Lipofectamine 3000 (Thermo Fisher Scientific) for 8 h prior to PEDV infection. For PEDV infection assays shown in Fig. S4C, organoid-derived monolayers were infected with PEDV ZJ08 at an MOI of 0.1 for 48 h. For PAMP stimulation assays shown in Fig. S4D through F, monolayers were transfected with Poly(I:C) or *in vitro*-transcribed 375-760 nt PEDV PAMP RNA using Lipofectamine 3000 for 8 h prior to PEDV infection, and cells and culture supernatants were collected at 24 h post-infection for RT-qPCR analysis and viral titration. Viral titers in organoid supernatants were determined by plaque assay using Vero E6 cells. Unless otherwise stated, experiments were performed using three independent organoid-derived monolayer cultures generated from separate organoid passages.

### Animals

Specific-pathogen-free 4–6-week-old female BALB/c mice were purchased from the Animal Research Center of Yangzhou University and housed in individually ventilated cages. Neonatal piglets (3 days old) were obtained from a cesarean-derived, colostrum-deprived herd maintained by Peiqi Agricultural and Animal Husbandry Technology Co., Ltd. (Jiangsu, China), and were confirmed to be seronegative for major swine pathogens, including PEDV, TGEV, and PDCoV. To eliminate potential maternal antibody interference, piglets were artificially fed throughout the study. All animals were of comparable body weight and were acclimatized for at least 24 h before experimentation to minimize stress. All animal studies were conducted in accredited, specific-pathogen-free (SPF) facilities and in accordance with national guidelines for the care and use of laboratory animals. Randomization and blinded evaluation were incorporated where applicable to minimize experimental bias.

### scRNA-seq analysis of intestinal responses to PEDV infection

To characterize intestinal transcriptional responses to PEDV infection, we analyzed the publicly available scRNA-seq data set GSE175411 from NCBI GEO. This data set contains jejunal cells collected 24 h after oral PEDV challenge in 3-day-old piglets, and the infecting PEDV strain (AH2012/12, GenBank KU646831) shares > 95% nucleotide identity with the strain used in our study. Age-matched and time-matched healthy controls are included, making this data set highly compatible with our experimental conditions. Raw FASTQ files were processed with Cell Ranger (10× Genomics, v3.1.0) using the Sscrofa11.1 reference genome. Downstream analyses were performed in Seurat v3. Cells with <500 UMIs or >15% mitochondrial transcripts were removed, and log-normalized expression matrices were used for subsequent analyses.

We first performed KEGG enrichment analysis across all intestinal mucosal cell types to define global pathways altered by PEDV infection. To assess innate immune activation at the cell-type level, KEGG enrichment was conducted separately in epithelial, T, B, and myeloid cells, focusing on pathways such as RIG-I-like receptor signaling, NF-κB activation, cytokine–receptor interactions, and interferon-stimulated gene (ISG) responses. For epithelial cells, we further examined RIG-I downstream effectors (e.g., MAVS–TBK1–IRF3) and representative ISGs and generated heatmaps comparing expression patterns between control and infected groups. KEGG enrichment was performed using clusterProfiler, with adjusted *P* < 0.05 as the significance threshold. Gene annotations were obtained from org.Ss.eg.db, and data visualization, including bubble plots and heatmaps, was performed using ggplot2 and pheatmap.

### RNA extraction and quantitative RT-PCR

Total RNA from tissues and cultured cells were extracted using TRIzol Reagent following the manufacturer’s protocol. For tissue samples, approximately 30–50 mg of material was homogenized in 1 mL of TRIzol using stainless-steel grinding beads on a mechanical disruptor. For cell samples, cultured cells were lysed directly in TRIzol after removing the culture medium. RNA quantity and purity were assessed by spectrophotometry. First-strand cDNA was synthesized from 500 ng to 1 μg of total RNA using HiScript III RT SuperMix (Vazyme, Nanjing, China). Quantitative PCR was performed using a SYBR Green–based qPCR Master Mix (Vazyme) on an Applied Biosystems real-time PCR platform. Each reaction was run in technical triplicate, and melt-curve analysis was included to confirm amplification specificity. GAPDH served as the internal reference gene, and relative transcript levels were calculated using the 2^−ΔΔCT^ method. All primer sequences used in this study are provided in Table S1.

### Western blot analysis

Tissue and cell samples were lysed in ice-cold RIPA buffer supplemented with PMSF. Lysates were clarified by centrifugation, and the supernatants were mixed with loading buffer and denatured at 100°C for 15 min. Equal amounts of protein were separated on 10% SDS–PAGE gels and transferred onto 0.22 μm PVDF membranes (Millipore, USA). Membranes were blocked with 5% skim milk for 2 h at room temperature, followed by overnight incubation at 4°C with the indicated primary antibodies, including monoclonal antibodies against the N proteins of TGEV, PDCoV, and PEDV, and anti-H1N1-HA, as well as RIG-I. For the detection of tagged proteins, mouse anti-Flag monoclonal antibodies were employed. After washing, membranes were incubated with HRP-conjugated goat anti-mouse IgG or goat anti-rabbit IgG secondary antibodies for 2 h at room temperature. Protein bands were visualized using enhanced chemiluminescence (ECL; Biosharp, Hefei, China) and captured with a digital imaging system. Band intensities were quantified using ImageJ software, and relative protein levels were normalized to GAPDH.

### Tissue processing, *in situ* hybridization, and immunofluorescence

Small intestinal tissues were fixed in 4% paraformaldehyde for 24 h, dehydrated through a graded ethanol series, cleared in xylene, and embedded in paraffin. Paraffin blocks were sectioned at a thickness of 5 μm using a rotary microtome (Leica, Germany), mounted on glass slides, and dried at 37°C. For RNA *in situ* hybridization, sections were dewaxed, rehydrated, and treated with proteinase K for antigen retrieval, followed by denaturation at 78°C, dehydration, and overnight hybridization at 37°C with RIG-I–specific probes (2 μM) designed based on the porcine RIG-I sequence (GenBank accession no. MF358966). For immunofluorescence staining, sections were dewaxed, rehydrated, permeabilized with Triton X-100, and blocked with 5% BSA, then incubated with primary antibodies at 4°C overnight and subsequently with fluorophore-conjugated secondary antibodies for 2 h at 37°C in the dark. Nuclei were counterstained with DAPI, slides were mounted with antifade medium, and images were acquired using a Zeiss Axio Observe.

### Plasmid construction and transfection

Full-length RIG-I cDNA was amplified by PCR and inserted into the pLVX-3Flag-MCS-P2A vector via homologous recombination. For knockdown experiments, three shRNA sequences targeting RIG-I were designed, annealed, and cloned into the pLVX-shRNA3 vector backbone, whereas MDA5-specific siRNA oligonucleotides were synthesized for transient silencing. The expression vector (pLVX-3Flag-MCS-P2A), shRNA3 backbone, and lentiviral packaging plasmids (pLP1, pLP2, and pLP-VSVG) were preserved in our laboratory. Primer sequences for RIG-I cDNA amplification and shRNA construction are listed in Table S1. All plasmid constructs were verified by Sanger sequencing and prepared using an EndoFree Plasmid Kit (OMEGA, USA). Lentiviral particles were generated by co-transfecting HEK293T cells with the expression or shRNA plasmids together with the Lenti-X packaging plasmids (pLP1, pLP2, and pLP-VSVG) using Lipofectamine 3000 (Thermo Fisher Scientific). The transfection mixture contained 3 μg expression or shRNA plasmid, 1.95 μg pLP1, 0.75 μg pLP2, 1.05 μg pLP-VSVG, and P3000 reagent in Opti-MEM. Lip3000 reagent was diluted in Opti-MEM and mixed with DNA, followed by 20 min incubation at room temperature. Viral supernatants were harvested at 48 h and 72 h post-transfection, clarified by centrifugation at 12,000 rpm for 30 min, and filtered through 0.45 μm membranes. Viral particles were then concentrated by ultracentrifugation at 30,000 rpm for 2 h at 4℃, resuspended in pre-chilled PBS, and filtered through 0.22-μm membranes before storage at −80℃. At 48 h post-infection, puromycin selection was applied to establish stable RIG-I overexpression and RIG-I knockdown cell pools. Cells were maintained under puromycin until >80% of cells exhibited stable expression or knockdown, and puromycin concentration was adjusted when excessive cell death occurred to maintain viability. The resulting RIG-I–overexpressing and shRNA-mediated RIG-I–knockdown stable cell lines were expanded and maintained in puromycin-free medium for subsequent experiments.

### Virus titer determination

Virus titers were determined by the plaque-forming unit (PFU) assay. Vero cells were seeded into 12-well plates and cultured at 37°C until reaching approximately 90% confluence. Serial gradient dilutions of virus suspensions were added to the monolayers (1 mL/well) and incubated for 1 h at 37°C to allow adsorption. After removal of the inoculum, the cells were overlaid with DMEM containing 2% FBS and 1% low-melting-point agarose. Following incubation at 37°C for 48 h, the cells were fixed with 4% paraformaldehyde for 30 min, the agar overlay was removed, and plaques were visualized by staining with 1% crystal violet. Viral titers (PFU/mL) were calculated based on plaque numbers and the dilution factor.

### RNA immunoprecipitation and sequencing (RIP-seq)

MARC-145 cells were grown to 60–70% confluence and infected with PEDV at 1 MOI. At 24 h post-infection, cells were harvested and lysed in RIP lysis buffer supplemented with protease and RNase inhibitors. Genomic DNA was removed by DNase I digestion, and lysates were clarified by centrifugation to obtain the soluble fraction for immunoprecipitation. RIG-I–RNA complexes were isolated using a RIP-seq kit (Bersinbio, Guangzhou, China) following the manufacturer’s instructions. Briefly, clarified lysates were incubated overnight at 4°C with magnetic beads conjugated to an anti-RIG-I monoclonal antibody. Beads were washed repeatedly under gentle conditions to minimize nonspecific binding. RNA-protein complexes were eluted by proteinase K digestion, and RNA was purified with TRIzol. Both RIP and input samples were assessed for RNA quality and integrity to ensure suitability for downstream sequencing.

For library construction, high-quality RNA was fragmented to generate templates of appropriate size for cDNA synthesis. Libraries were evaluated for quality control and subjected to high-throughput sequencing by Guangzhou Genedenovo Biotechnology Co., Ltd. Raw reads were processed to remove adapters, poly(A) sequences, and low-quality bases, yielding clean reads with Q20 and Q30 values exceeding 90%, meeting the requirements for next-generation sequencing analyses. The actual value of Q20 was 4,856,684,292 bp (97.61%), and the actual value of Q30 was 4,672,784,891 bp (93.91%).

### *In vitro* RNA synthesis

Genomic RNA of PEDV was extracted and reverse-transcribed into cDNA. Target fragments were amplified by PCR using primers containing a T7 promoter sequence at the 5′ end (primer sequences listed in Table S1). Purified linear PCR products served as templates for *in vitro* transcription. RNA was synthesized using T7 RNA polymerase according to the manufacturer’s protocol (Vazyme, Nanjing, China) at 37°C for 2 h, followed by DNase I treatment and TRIzol purification. The 375–760 nt fragment corresponding to the key PEDV PAMP was generated by *in vitro* transcription. The 717–744 nt binding fragment and its 740-site mutant were chemically synthesized (Hippo Biotechnology Co., Ltd., China). RNAs were transfected into cells using Lipofectamine 3000, and innate immune activation was quantified by RT-qPCR.

### Preparation of viral RNA fragments

RNA pull-down assays were performed using the Pierce Magnetic RNA-Protein Pull-Down Kit (Thermo Fisher Scientific, USA). Synthesized RNAs were first labeled with desthiobiotin using T4 RNA ligase at 16°C, followed by purification through chloroform extraction and ethanol precipitation. Biotin-labeled RNAs were incubated with streptavidin magnetic beads at room temperature to immobilize the RNA. RNA-bound beads were then incubated overnight at 4°C with whole-cell lysates prepared from HEK293T cells transiently expressing full-length RIG-I or its truncated mutants. After extensive washing, bound proteins were eluted at 37°C using the kit’s elution buffer. The eluates were collected and analyzed by Western blotting to identify proteins specifically interacting with the RNA.

### Molecular docking and dynamics simulations

Molecular docking and molecular dynamics (MD) simulations were conducted by Tianji-Suan Technology Co., Ltd. The porcine RIG-I protein sequence was obtained from the UniProt database, and its three-dimensional structure was predicted using AlphaFold3. The structural model was energy-minimized with the Rosetta Relax module before subsequent analyses. RNA structures were also generated using AlphaFold3. Protein–RNA docking was initially performed with HDOCK (rigid docking), followed by refinement with RosettaDock (flexible docking), and candidate complexes were ranked using the Rosetta scoring function. Protein–RNA interactions were further analyzed with LigPlot, while overall binding energies were assessed using the Rosetta Interface_analyzer module. PyMOL was used to visualize the docked complexes. MD simulations were carried out with Gromacs 2023.3 using the AMBER force field for both protein and RNA molecules. To evaluate the contribution of individual nucleotides, the top 10 residues predicted to participate in RIG-I binding were subjected to *in silico* saturation mutagenesis using FoldX 5.1. At each nucleotide position, the original base (A, C, G, or U) was systematically substituted with the other three bases, thereby generating all possible single-nucleotide variants. The effect of each mutation on complex stability was quantified by calculating the binding free energy difference (ΔΔG bind = ΔG bind[mutant] – ΔG bind[wild type]).

### Evaluation of broad-spectrum antiviral activity

To assess the impact of PEDV-derived PAMP RNA on viral replication, MARC-145, ST, and A549 cells were transfected with *in vitro*–transcribed PAMP RNA using Lipofectamine 3000. Following transfection, cells were infected with PEDV, TGEV, PDCoV, or H1N1 (MOI = 0.1) under standard culture conditions. After 24 h of infection, total RNA and protein were harvested. The transcriptional levels of host innate immune genes (RIG-I and IFN-β) and viral genes (PEDV N, TGEV N, PDCoV N, and H1N1 HA) were quantified by RT-qPCR, while the corresponding viral proteins were analyzed by Western blotting.

Twelve healthy 3-day-old Duroc/Landrace/Yorkshire piglets were obtained from Jiangsu Nanjing Pig Farm (Nanjing, China), with negative serology for porcine reproductive and respiratory syndrome virus, PDCoV, PEDV, and TGEV. All piglets had unrestricted access to water and food. Twelve piglets were randomly assigned to four groups (*n* = 3 per group): mock, PBS, Poly(I:C), and PAMP RNA. PAMP RNA was synthesized *in vitro* and delivered intramuscularly using a polyethyleneimine (PEI; Meryer, Shanghai, China)–based nucleic acid delivery system. Piglets in the mock and PBS groups received PEI alone or PEI mixed with PBS, whereas those in the Poly(I:C) and PAMP groups were injected with 100 µL PEI containing 100 μg Poly(I:C) or PAMP RNA, respectively. At 24 h post-injection, all groups except mock were orally challenged with PEDV (10⁷ PFU). Clinical signs, including vomiting and diarrhea, were monitored, and at 36 h post-infection, piglets were humanely euthanized. Jejunal and ileal tissues were collected for histopathology, immunofluorescence, and molecular analyses. Viral replication and host innate immune responses were evaluated by RT-qPCR, Western blotting, and serum ELISA.

### Effect of PEDV PAMP on innate immune activation

To investigate the innate immune effects of PEDV-derived PAMP RNA, mice and neonatal piglets were used as *in vivo* models. Thirty 4- to 6-week-old SPF BALB/c female mice (*n* = 5 per group) were randomly assigned to receive vehicle control, Poly(I:C), or PEDV PAMP RNA formulated with jetRNA (Polyplus, France), administered via intramuscular or intravenous injection. The jetRNA served as the *in vivo* transfection reagent. Each injection contained 10 µL of jetRNA and 10 µg of sample prepared as a 200 µL dilution. The 10 μg dose was selected based on published *in vivo* RNA delivery studies and preliminary optimization experiments, which indicated efficient innate immune activation without observable adverse effects (44). At 24 h post-treatment, blood, spleen, and lung samples were collected to assess cytokine induction and innate immune gene expression. Nine 3-day-old Duroc/Landrace/Yorkshire piglets were randomly assigned to three groups (*n* = 3 per group) and received intramuscular injection of vehicle, PEDV PAMP RNA, or Poly(I:C). Piglets in the PEDV PAMP and Poly(I:C) groups each received 100 μg RNA formulated with polyethyleneimine (PEI; Meryer, Shanghai, China), with the dose selected by body weight-adjusted scaling from the mouse experiments. At 24 h post-injection, jejunal and ileal tissues were collected for molecular analyses. Host innate immune responses were evaluated by RT-qPCR and serum ELISA.

### Effect of PEDV PAMP on adaptive immune activation

To evaluate whether PEDV-derived PAMP RNA enhances adaptive immune responses, a murine influenza vaccination and challenge model was established. Fifty 4-week-old mice were randomly allocated into five groups (*n* = 10 per group). Animals were immunized intramuscularly with inactivated H1N1 vaccine alone or in combination with PAMP RNA or Poly(I:C). In the combination groups, 10 μg PEDV PAMP RNA or 10 μg Poly(I:C) per mouse was administered, matching the dose used in the innate immune activation assay. Immunizations were administered every two weeks, and blood samples were collected from the retro-orbital sinus one week after each immunization to determine serum antibody levels by ELISA. Ten days after the booster immunization, mice were challenged intranasally with an equal dose of H1N1 virus (10^6^ EID₅₀). The mock group served as a negative control. Clinical symptoms, body weight, and survival were monitored throughout the experiment. A subset of mice was euthanized on day 6 post-challenge for viral load quantification and histopathological examination of lung tissues.

### Clinical and histopathological scoring

Clinical severity of diarrhea in piglets was evaluated by a standardized fecal consistency scoring system adapted from previous studies on porcine enteric disease models. Piglet feces were observed and scored daily based on visual stool morphology and water content: 1 = normal, well-formed feces; 2 = soft, non-formed feces; 3 = paste-like or semi-liquid; 4 = watery diarrhea; and 5 = hemorrhagic or necrotic diarrhea (45). Higher scores indicate more severe diarrhea and intestinal dysfunction.

For histopathological assessment of mouse lungs, a semi-quantitative scoring system was used to evaluate lesion severity across multiple parameters following established approaches for pulmonary disease models (46). Lung sections stained with hematoxylin and eosin were examined by a blinded pathologist, and the following features were scored: alveolar wall thickening (0–3), inflammatory cell infiltration (0–3), alveolar atelectasis (0–3), edema and exudation (0–3), and bronchial epithelial necrosis (0–3). The total histopathology score (0–15) was calculated as the sum of individual parameter scores, providing an integrated measure of lung injury severity.

### Statistical analysis

All data are presented as mean ± standard deviation (SD). Statistical analyses were performed using SPSS version 17.0 (SPSS Inc., Chicago, IL, USA). For comparisons involving more than two groups, one-way ANOVA was conducted, followed by Fisher’s least significant difference (LSD) post-hoc test when the overall F-test indicated significance. For comparisons between two groups, an unpaired two-tailed Student’s *t*-test was applied. Statistical significance was defined as follows: ns, not significant; *P < 0.05, significant; **P < 0.01, highly significant; and ***P < 0.001, extremely significant. Unless otherwise specified, all experiments were independently repeated at least three times.

## Data Availability

The scRNA-seq dataset generated from PEDV-infected piglet jejunum is publicly available in the NCBI GEO database (accession number GSE175411). The RIP-seq data used in this study are provided in Data S1. The authors confirm that all data supporting the findings of this work are available within the paper and its supplemental material and are fully accessible without restriction.

## SUPPLEMENTAL MATERIAL

Data S1 jvi.00483-26-s0001.xlsx RIG-I-associated enrichment peaks across the PEDV genome.

Supplemental figures jvi.00483-26-s0002.pdf Fig. S1 to S8.

Table S1 jvi.00483-26-s0003.docx Primer sequences used for experiments.

ASM does not own the copyrights to Supplemental Material that may be linked to, or accessed through, an article. The authors have granted ASM a non-exclusive, world-wide license to publish the Supplemental Material files. Please contact the corresponding author directly for reuse.
