# Supplementary material for: RIG-I–dependent sensing of PEDV shapes epithelial antiviral immunity in the intestinal mucosa
Source: J Virol. 2026 Jun 16;100(7):e00483-26. doi: 10.1128/jvi.00483-26 (PMC13386879; doi:10.1128/jvi.00483-26)
Supplement: Supplemental figures — Fig. S1 to S8. [file jvi.00483-26-s0002.pdf]

Supporting information

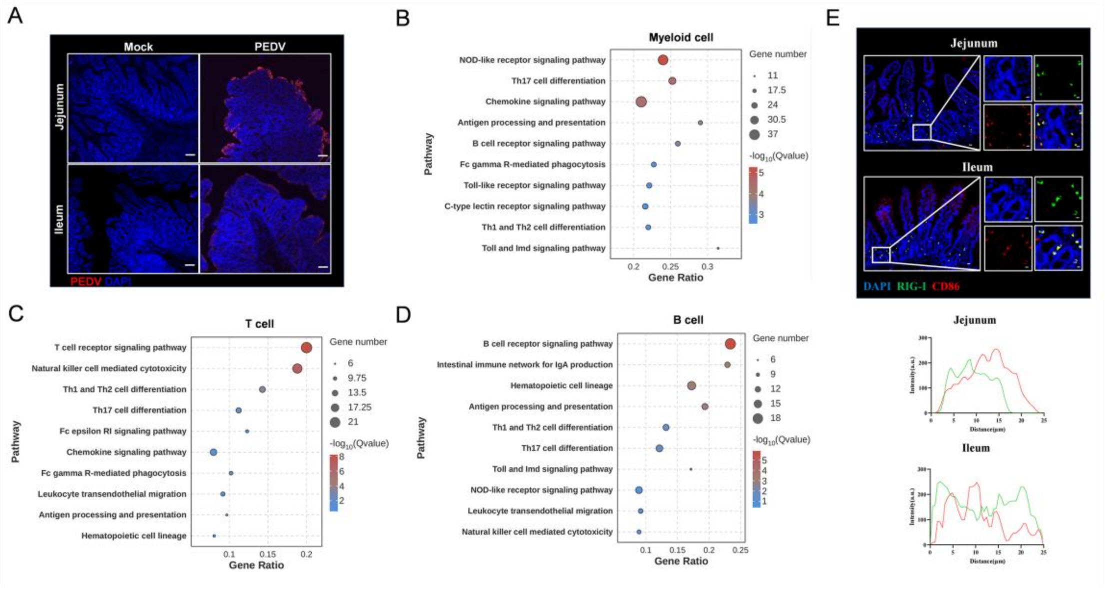

Fig S1. Immune pathway activation across intestinal mucosal cell subsets following oral PEDV infection

(A) Immunofluorescence staining of jejunum and ileum sections from mock- and PEDV-infected piglets showing strong PEDV antigen signals (red) localized along the villus epithelium. Nuclei are counterstained with DAPI (blue). Scale bars, 100 μm. (B–D) KEGG pathway enrichment analysis of differentially expressed genes identified by scRNA-seq in intestinal myeloid cells (B), T cells (C), and B cells (D) after oral PEDV infection. Enriched pathways include innate immune signaling (e.g., NOD-like receptor, Toll-like receptor, FcγR-mediated phagocytosis), antigen presentation, leukocyte activation, and adaptive immune pathways such as Th17/Th1/Th2 differentiation and B cell receptor signaling. (E) FISH shows the spatial distribution of RIG-I transcripts (green) and the myeloid/antigen-presenting cell marker CD86 (red) in jejunal and ileal mucosa. Nuclei are counterstained with DAPI (blue). Enlarged insets highlight regions of interest, revealing that RIG-I transcripts are predominantly localized in $CD86^{+}$myeloid cells within the lamina propria, with minimal RIG-I signal detected in epithelial cells. Line-scan analysis further demonstrates that RIG-I and CD86 signals show spatial adjacency or partial overlap, reflecting localized innate immune activation in the lamina propria. Data

represent mean ± SD from at least three biological replicates.

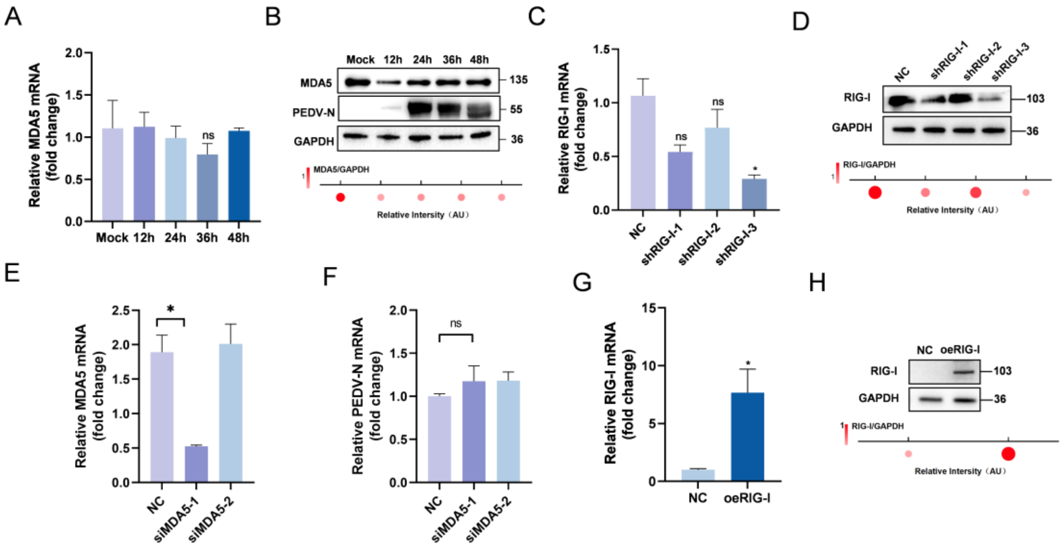

Fig S2. Functional comparison of RIG-I and MDA5 in PEDV infection by knockdown analyses and RIG-I overexpression

(A) Time-course RT-qPCR quantification of MDA5 mRNA in PEDV-infected MARC-145 cells from 6 to 48 hpi. (B) Western blot analysis of MDA5 protein expression at corresponding time points with bubble-plot summarization of band intensities. (C–D) Evaluation of RIG-I knockdown efficiency using three shRNA constructs: RT-qPCR analysis of RIG-I transcript levels (C) and Western blot measurement of RIG-I protein (D). (E–F) Functional consequence of MDA5 silencing during PEDV infection. RT-qPCR assessment of MDA5 knockdown efficiency (E) and quantification of PEDV N RNA levels (F) in siRNA-transfected MARC-145 cells. (G–H) Verification of RIG-I overexpression, assessed by RT-qPCR measurement of RIG-I mRNA (G) and Western blot confirmation of RIG-I protein expression (H). Data are presented as mean ± SD from at least three independent experiments. Statistical significance was assessed using Student’s t-test for two-group comparisons and one-way ANOVA for multiple-group comparisons (**P* < 0.05; ns, not significant).

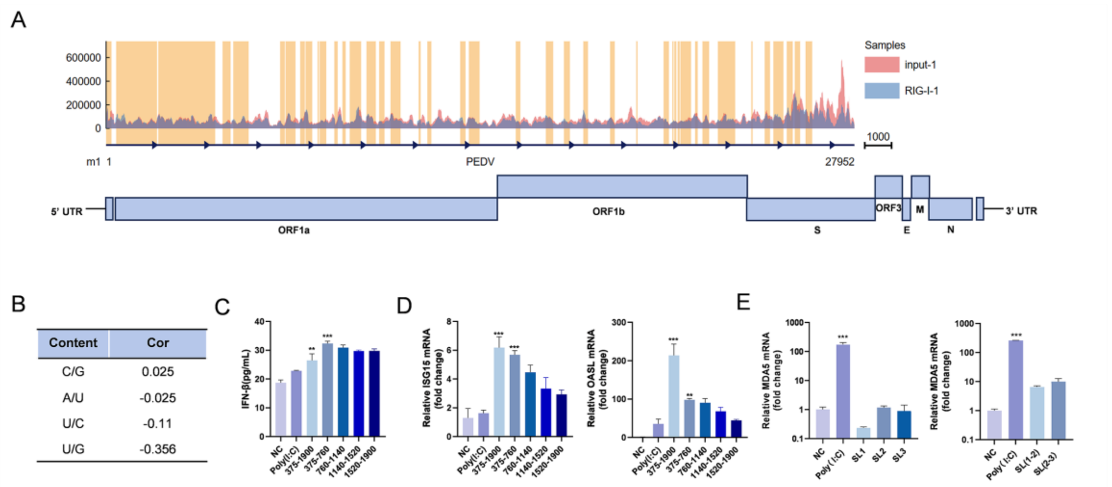

Fig S3. Structural and sequence determinants of RIG-I recognition within the PEDV genome

(A) RIP-seq read coverage of input and RIG-I immunoprecipitated RNA across the PEDV genome, showing enrichment within the 5′ ORF1a region. (B) Pairwise nucleotide-content correlation analysis of enriched RIG-I-associated PEDV RNA regions. (C–D) Functional assessment of truncated PEDV RNA fragments transfected into MARC-145 cells. IFN-β secretion was measured by ELISA (C), and ISG15 and OASL mRNA expression was quantified by RT-qPCR (D). (E) RT-qPCR analysis of MDA5 mRNA expression in MARC-145 cells transfected with single or combined stem-loop RNA structures derived from the 375–760 nt region. These structures did not significantly induce MDA5 mRNA expression under the tested conditions. Data are presented as mean ± SD from at least three independent experiments. Statistical significance was determined by one-way ANOVA.

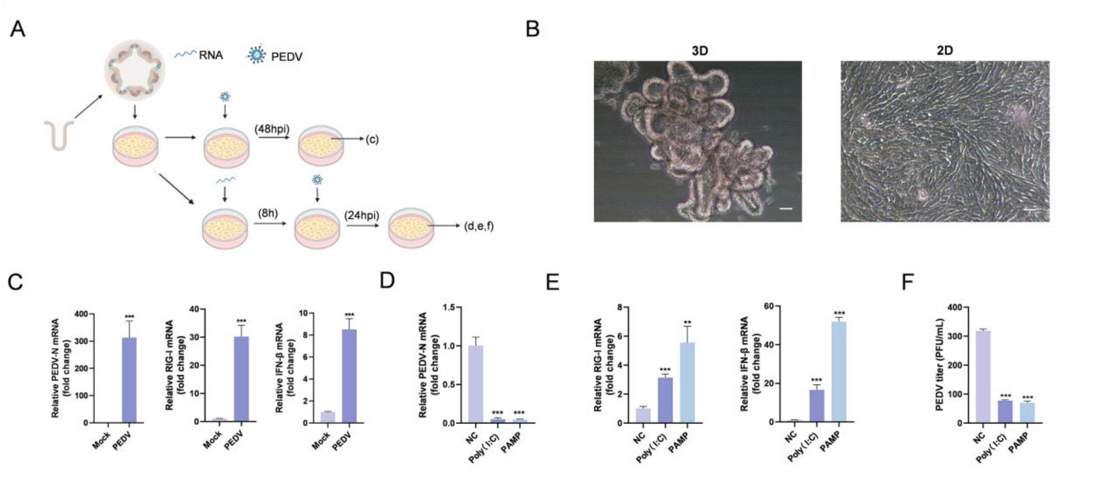

Fig. S4. Validation of PEDV PAMP-mediated RIG-I activation and antiviral

activity in porcine intestinal organoid-derived epithelial monolayers

(A) Schematic of 3D porcine intestinal organoid culture, generation of 2D epithelial monolayers, PEDV infection, RNA transfection, and downstream analyses. (B) Representative images of 3D organoids and organoid-derived 2D epithelial monolayers. Scale bars, 25 μm. (C) RT-qPCR analysis of PEDV N, RIG-I, and IFN-β mRNA expression in organoid-derived monolayers following PEDV infection. (D–E) Organoid-derived monolayers were transfected with Poly(I:C) or the 375–760 nt PEDV PAMP RNA before PEDV infection. PEDV N mRNA levels (D) and RIG-I/IFN-β mRNA expression (E) were quantified by RT-qPCR. (F)Viral titers in culture supernatants were determined by plaque assay using Vero E6 cells. NC refers to PEDV-infected monolayers treated with transfection reagent alone. Data are presented as mean ± SD from three independent organoid-derived monolayer cultures generated from separate organoid passages. Statistical significance was determined by one-way ANOVA.

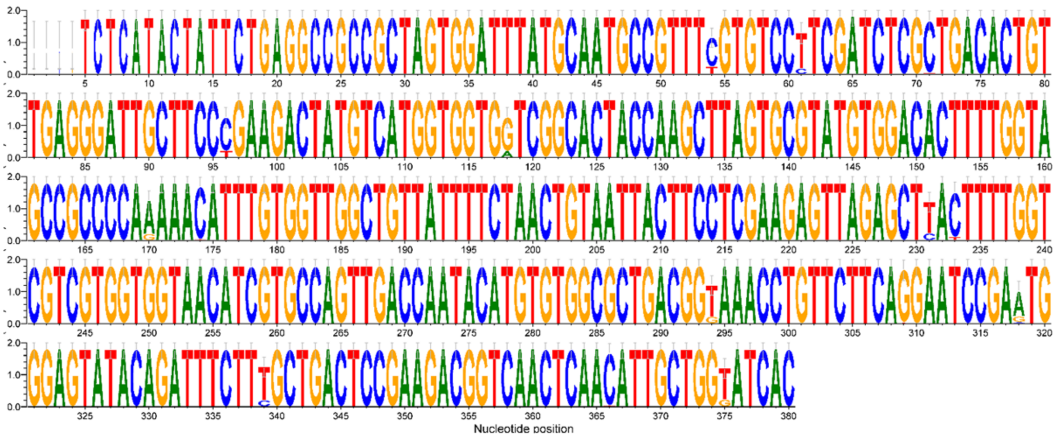

Fig. S5. Sequence conservation of the PEDV 375–760 nt PAMP region among representative PEDV strains.

The 375–760 nt region was extracted according to the PEDV ZJ08 genome coordinates and aligned with corresponding regions from 44 representative PEDV complete genome sequences. Sequence conservation was visualized using WebLogo. Dominant nucleotides are present at most positions, with limited polymorphic sites across the analyzed strains.

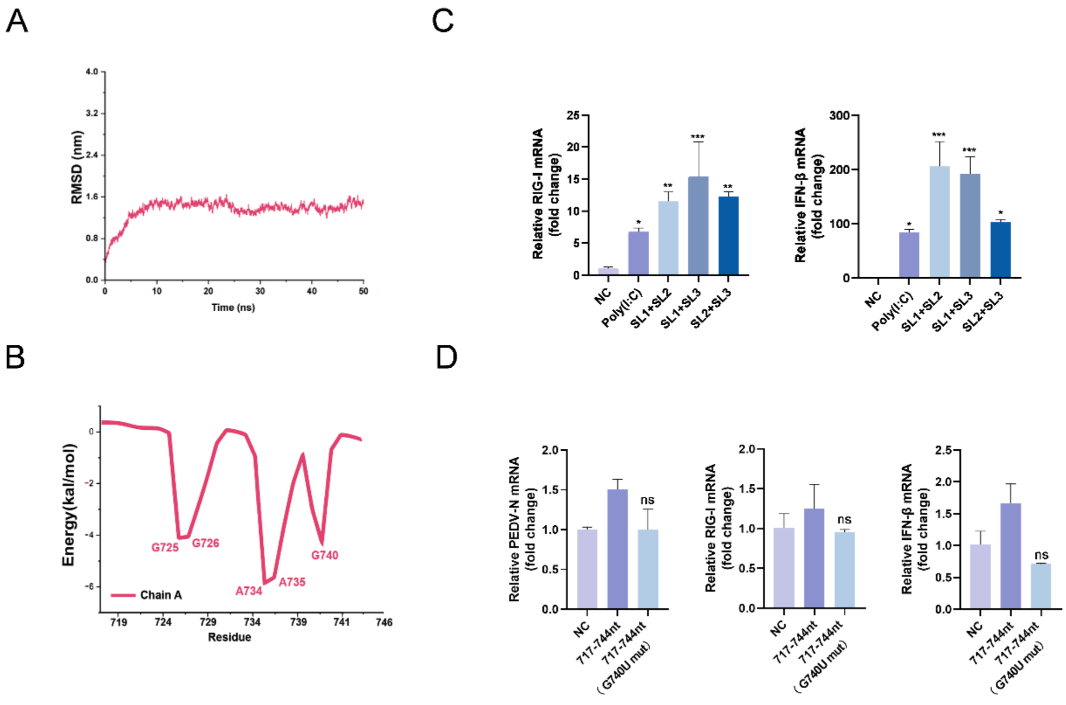

Fig S6. Structural determinants of RIG-I binding and activation by PEDV RNA fragments

(A) RMSD plot of the RIG-I–PEDV 717–744 nt RNA complex during a 50-ns molecular dynamics simulation. (B) Per-nucleotide binding free-energy decomposition of the 717–744 nt RNA fragment, highlighting nucleotides predicted to contribute strongly to RIG-I interaction. (C) RT-qPCR analysis of RIG-I and IFN-β mRNA expression in MARC-145 cells transfected with paired stem-loop constructs, showing that combined stem-loop presentation enhances innate immune activation compared with NC. (D) RT-qPCR analysis of PEDV N, RIG-I, and IFN-β mRNA expression in MARC-145 cells transfected with the wild-type 717–744 nt RIG-I-binding fragment or its G740U mutant followed by PEDV infection. Neither the wild-type 717–744 nt fragment nor the G740U mutant significantly altered these downstream readouts under the tested conditions. Data are presented as mean ± SD from at least three independent experiments. Statistical significance was determined by one-way ANOVA.

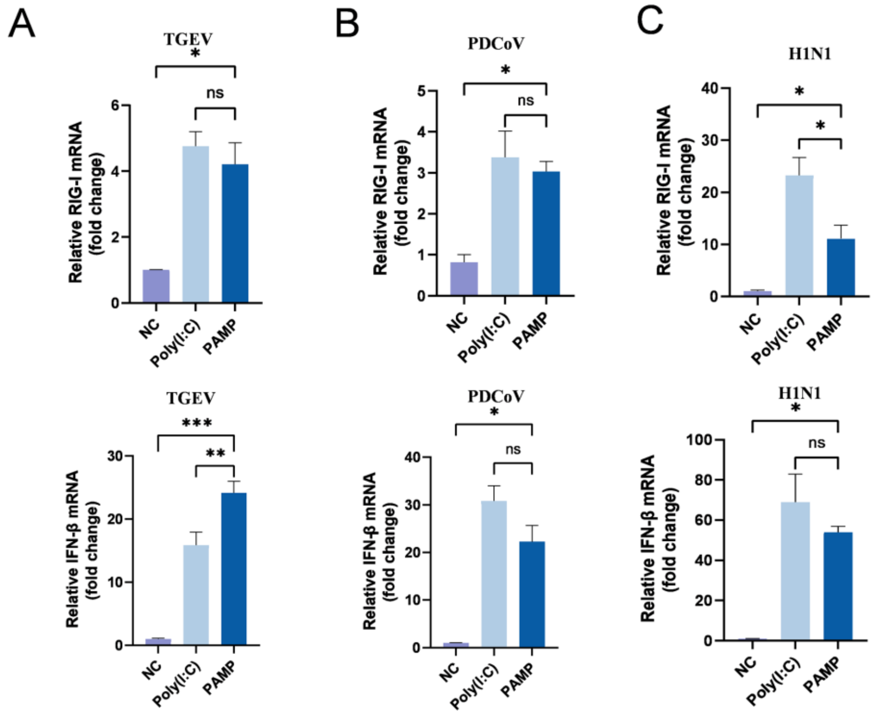

Fig S7. PEDV-derived PAMP RNA induces robust RIG-I signaling across diverse viral infections

(A–B) RT-qPCR analysis of RIG-I and IFN-β mRNA expression in porcine ST cells transfected with NC, Poly(I:C), or PEDV PAMP RNA prior to infection with TGEV (A) or PDCoV (B). (C) RT-qPCR analysis of RIG-I and IFN-β mRNA expression in human A549 cells transfected with NC, Poly(I:C), or PEDV PAMP RNA prior to H1N1 infection. PAMP RNA significantly enhances innate immune activation compared with controls across all tested viral infections. Data are presented as mean ± SD from at least three biological replicates. Statistical significance was assessed using one-way ANOVA (*$P < 0.05$, **$P < 0.01$, ***$P < 0.001$; ns, not significant).

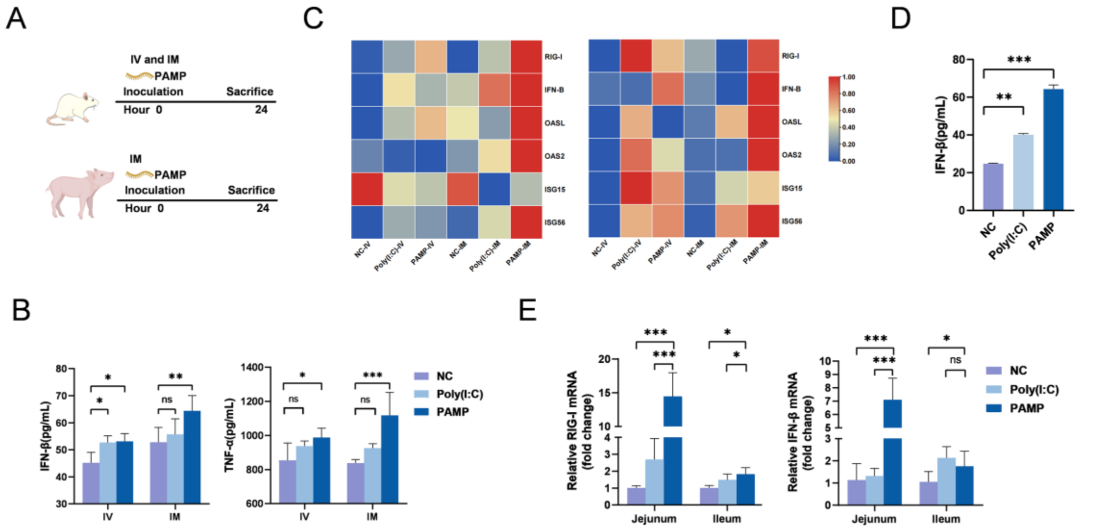

Fig S8. PEDV-derived PAMP RNA enhances transcription of RIG-I downstream antiviral effectors *in vivo*

(A) Schematic diagrams of PAMP RNA administration in mice (n = 5 per group) and piglets (n = 3 per group), illustrating experimental design and delivery routes. (B) ELISA-based quantification of serum IFN-β and TNF-α levels in mice following PAMP RNA administration, demonstrating systemic innate immune activation. (C) *In vivo* delivery of PAMP RNA using the jetRNA system in mice, followed by assessment of RIG-I–mediated innate immune activation in spleen and lung tissues. RT-qPCR results of RIG-I and downstream antiviral genes are presented as a heatmap, demonstrating robust activation of systemic and respiratory mucosal innate immunity. (D) Systemic and intestinal mucosal immune responses following PAMP administration in piglets. Serum IFN-β concentrations were measured by ELISA. (E) RIG-I and IFN-β expression in jejunum and ileum tissues of piglets was quantified by RT-qPCR, indicating strong PAMP-induced antiviral activation in intestinal mucosa. Data represent mean ± SD from at least three biological replicates. Statistical significance was evaluated using one-way ANOVA (*$P < 0.05$, **$P < 0.01$, ***$P < 0.001$; ns, not significant).

Table S1. Primer sequences used for experiments

Data S1. RIG-I-associated enrichment peaks across the PEDV genome
