# Supplementary material for: RIG-I–dependent sensing of PEDV shapes epithelial antiviral immunity in the intestinal mucosa
Source: J Virol. 2026 Jun 16;100(7):e00483-26. doi: 10.1128/jvi.00483-26 (PMC13386879; doi:10.1128/jvi.00483-26)
Supplement: Table S1 — Primer sequences used for experiments. [file jvi.00483-26-s0003.docx]

**S1 Table. Primer sequences used for experiments**

| **Gene** | **Primer** | **Sequence (5'-3')*** |
| --- | --- | --- |
| **For *in vitro* and *in vivo* detection of immune activation RT-qPCR experiments** | | |
| pRIG-I | Forward | CTGGAGCTTGCTTTACCT |
|  | Reverse | CCTTCCCCTTTCGTCCTTGT |
|  | Probe | FAM-AGUGAUGGAAUUGUCCCAUUGGUAAGACAGUUCACAAGAAU |
| mRIG-I | Forward | GAATGCCACAACACCAGT |
|  | Reverse | CATCAAGAGAAGCACACAG |
| pMDA5 | Forward | GTCCGAGACGTCCAGACT |
|  | Reverse | CACTTGCCCGCGAATTAACA |
| mMDA5 | Forward | GAAATCGCAAAGACCGT |
|  | Reverse | CAATACTCATCATCACCACC |
| pIFN-β | Forward | GCTAACAAGTGCATCCTC |
|  | Reverse | CCAGGAGCTTCTGACATGCC |
| mIFN-β | Forward | CCTGGAGCAGCTGAATGG |
|  | Reverse | AGGCACAGTGACTGTACT |
| pOASL | Forward | GGCTGACCCCACCTACAA |
|  | Reverse | GGGACTGGGCTCTTGTTGTT |
| mOASL | Forward | GAGGACCTGGTCAAGGT |
|  | Reverse | CACAGCCACACACAGCTTC |
| pOAS2 | Forward | AAACTGGGGGTCCCATCT |
|  | Reverse | GTCCACGGTCTGGTCGATTT |
| mOAS2 | Forward | CAGGTGCTGAAGGACATC |
|  | Reverse | TCCAGGTAGCCATCGTCTTC |
| pISG15 | Forward | CTATGAGGTCTGGCTGAC |
|  | Reverse | ACGGTGCACATAGGCTTGA |
| mISG15 | Forward | CAGCATCCTGGTGGAAG |
|  | Reverse | TGCGGTCAGGGTTCTCTTA |
| pISG56 | Forward | TCCGACACGCAGTCAAGT |
|  | Reverse | TGTAGCAAAGCCCTGTCTGG |
| mISG56 | Forward | GAGGCTACAGCCACCTAC |
|  | Reverse | CAGGTCCAGGTGGTCTTCA |
| mouseβ-actin | Forward | CAATTCCCGTGGTCG |
|  | Reverse | TTTACGTTGGCCCTTCA |
| mouseRIG-I | Forward | ATGTGCCCCTACTGGTTGT |
|  | Reverse | CCCCAGAAATGCTCGC |
| mouseOASL | Forward | TTACCTCCTTCCCGACACCA |
|  | Reverse | CACCTTGGAGACCCTCACTG |
| mouseOAS2 | Forward | TTACCCCCAAAGTACGCCCT |
|  | Reverse | TGCAGAGCTGCCGGTATTTTA |
| mouseISG15 | Forward | GCCTGGGACCTAAAGGTGAAG |
|  | Reverse | TCTTCTGGGCAATCTGCTTCTT |
| mouseISG56 | Forward | AGAATTGGCACAACAGGCCA |
|  | Reverse | CTGCTCTATGTGAGCCACGA |
| mouseIFN-β | Forward | CTACAGGGCGGACTTCAAGA |
|  | Reverse | AGTCTCATTCCACCCAGTGC |
| **For *in vitro* and *in vivo* antiviral experiments and detection of virus** | | |
| mGAPDH | Forward | ACATCATCCCTGCCTCTA |
|  | Reverse | CCTGCTTCACCACCTTCTTG |
| pGAPDH | Forward | TCATCATCTCTGCCCCTTC |
|  | Reverse | GTCATGAGTCCCTCCACGAT |
| PEDV N | Forward | CACCTCCTGCTTCAC |
|  | Reverse | AGCTCCACGACCCTGGTTAT |
| TGEV N | Forward | CAATTCCCGTGGTCG |
|  | Reverse | TTTACGTTGGCCCTTCACC |
| PDCoV N | Forward | AACCCCAACAATCCTA |
|  | Reverse | GAGCGCATCCTTAAGTCTC |
| H1N1 HA | Forward | TCCACCTACCAGTGCTGACCAAC |
|  | Reverse | TGCTCTTTCGGTCGGCTGCATA |
| **For constructing lentiviral plasmids** | | |
| pLVX-RIG-I | Forward | atgatgacgataaacgaattcATGACTACGGAGCAGCGGCGC |
|  | Reverse | gttgcgccggagcctggatccTAAAGGTTGGGGTTTCCGGAA |
| shRIG-I-1 | Forward | GATCCGCAGGTTATTCTGGACTTTATCGAAATAAAGTCCAGAATAACCTGCTTTTTG |
|  | Reverse | AATTCAAAAAGCAGGTTATTCTGGACTTTATTTCGATAAAGTCCAGAATAACCTGCG |
| shRIG-I-2 | Forward | GATCCGCCAGAATCTTAGTGAGAATTCGAAAATTCTCACTAAGATTCTGGCTTTTTG |
|  | Reverse | AATTCAAAAAGCCAGAATCTTAGTGAGAATTTTCGAATTCTCACTAAGATTCTGGCG |
| shRIG-I-3 | Forward | GATCCGCAAATCAGATCCCAGTATATCGAAATATACTGGGATCTGATTTGCTTTTTG |
|  | Reverse | AATTCAAAAAGCAAATCAGATCCCAGTATATTTCGATATACTGGGATCTGATTTGCG |
| pLVX-RIG-I-N | Forward | atgatgacgataaacgaattcATGACTACGGAGCAGCGGC |
|  | Reverse | gttgcgccggagcctggatccCTCCAAAGCAAGTTTCAAAGTTTTG |
| pLVX-RIG-I-H | Forward | atgatgacgataaacgaattcAAACACAATAATATGTGCTCCTACAG |
|  | Reverse | gttgcgccggagcctggatccCTTTTCCCTAAATACTGCTTCATCC |
| pLVX-RIG-I-C | Forward | atgatgacgataaacgaattcAAAACCAAAACCTGTACCTGATAAGG |
|  | Reverse | gttgcgccggagcctggatccTCATTTGGCCATTTCTGCTGG |
| **For *in vitro* transcription of RNA** | | |
| 375-760 nt | Forward | TAATACGACTCACTATAGGGCTCATACTATTCTGAGGC |
|  | Reverse | ACGGCTTCACTAGCAGTG |
| 760-1140 nt | Forward | TAATACGACTCACTATAGGGGCCTGGATTGTAGATCGA |
|  | Reverse | CCAGGCGTAGCAGAGCAT |
| 1140-1520 nt | Forward | TAATACGACTCACTATAGGGTTCTGTTGTGGTTACACG |
|  | Reverse | AAGCTGCACTTGCAAGGT |
| 1520-1900 nt | Forward | TAATACGACTCACTATAGGGGGGAGCAGCTTAAGGCTG |
|  | Reverse | TTCAACGCGCTTGGAAAC |
